# Poly(Anthraquinonyl Sulfide)/CNT Composites as High‐Rate‐Performance Cathodes for Nonaqueous Rechargeable Calcium‐Ion Batteries

**DOI:** 10.1002/advs.202200397

**Published:** 2022-03-20

**Authors:** Siqi Zhang, Youliang Zhu, Denghu Wang, Chunguang Li, Yu Han, Zhan Shi, Shouhua Feng

**Affiliations:** ^1^ State Key Laboratory of Inorganic Synthesis and Preparative Chemistry Jilin University Changchun 130012 P. R. China; ^2^ State Key Laboratory of Supramolecular Structure and Materials College of Chemistry Jilin University Changchun 130012 P. R. China; ^3^ Advanced Membranes and Porous Materials Center Physical Sciences and Engineering Division King Abdullah University of Science and Technology (KAUST) Thuwal 23955‐6900 Saudi Arabia

**Keywords:** calcium‐ion batteries, co‐intercalation, high‐rate‐performance, poly(anthraquinonyl sulfide) (PAQS)

## Abstract

Calcium‐ion batteries (CIBs) are considered as promising alternatives in large‐scale energy storage due to their divalent electron redox properties, low cost, and high volumetric/gravimetric capacity. However, the high charge density of Ca^2+^ contributes to strong electrostatic interaction between divalent Ca^2+^ and hosting lattice, leading to sluggish kinetics and poor rate performance. Here, in situ formed poly(anthraquinonyl sulfide) (PAQS)@CNT composite is reported as nonaqueous calcium‐ion battery cathode. The enolization redox chemistry of organics has fast redox kinetics, and the introduction of carbon nanotube (CNT) accelerates electron transportation, which contributes to fast ionic diffusion. As the conductivity of the PAQS is enhanced by the increasing content of CNT, the voltage gap is significantly reduced. The PAQS@CNT electrode exhibits specific capacity (116 mAh g^−1^ at 0.05 A g^−1^), high rate capacity (60 mAh g^−1^ at 4 A g^−1^), and an initial capacity of 82 mAh g^−1^ at 1 A g^−1^ (83% capacity retention after 500 cycles). The electrochemical mechanism is proved to be that the PAQS undergoes reduction reaction of their carbonyl bond during discharge and becomes coordinated by Ca^2+^ and Ca(TFSI)^+^ species. Computational simulation also suggests that the construction of Ca^2+^ and Ca(TFSI)^+^ co‐intercalation in the PAQS is the most reasonable pathway.

## Graphical abstract

Poly(anthraquinonyl sulfide)@CNT composite is reported as nonaqueous calcium‐ion battery (CIB) cathode. The introduction of carbon nanotube (CNT) improves conductivity and reaction kinetics of the PAQS. The demonstrated rate performance of PAQS@34%CNT surpasses reported inorganic cathode materials for nonaqueous CIBs. The electrochemical mechanism is proved to be that Ca^2+^ and Ca(TFSI)^+^ prefer to co‐intercalate in the PAQS.

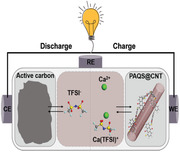

## 1 Introduction

The contradiction between the growing demand for renewable energy and depleted petrochemical resources has stimulated rapid development of cost‐effective and safe energy storage devices. Among them, multivalent ion‐based rechargeable batteries are of particular interest owing to multiple electrons’ reaction in comparison with the monovalent ions, theoretically beneficial to the high energy density. Ca‐ion batteries (CIBs) have low polarization and a lower standard potential (*E*_0_ = −2.87 V versus standard hydrogen electrode, SHE for Ca^2+^/Ca), which is only 0.17 V above that of lithium (*E*_0_ = −3.04 V versus SHE for Li^+^/Li), contributable to the widen voltage window.^[^ ^1^ ^]^ Moreover, the larger ion radius results in smaller charge density compared to that of Mg^2+^ and Zn^2+^. The cation–anion repulsive and cation–anion attractive forces are thus weaker, leading to an easier intercalation and diffusion of Ca^2+^.^[^ ^2^ ^]^ In addition, element Ca is a natural abundant element in nature, ranking the fifth in the earth's crust,^[^ ^3^ ^]^ making CIBs a good choice of environmental‐friendly, low‐cost, and sustainable energy storage device.

However, the development of CIBs is challenging because the reversible plating/stripping of metallic calcium only works with specific nonaqueous electrolyte.^[^ ^4^ ^]^ In addition, the deposition of calcium is accompanied with serious side reactions, such as continuous growing passivation film including CaF_2_, CaH_2_, CaCl_2_, and CaCO_3_, which render low Columbic efficiency.^[^ ^4^, ^5^ ^]^ The unsatisfactory performance of Ca‐metal anode drives the development for alternative anode materials. The limited property of Ca metal leads to difficultly in studying the electrochemical performance of electrode materials in a two‐electrode system.^[^ ^6^ ^]^ Therefore, a three‐electrode system and active carbon as counter electrode are usually applied for CIBs.^[^ ^7^ ^]^ Another challenge of CIBs is the lack of high‐performance electrode materials. Due to stronger electrostatic interaction between divalent Ca^2+^ and hosting lattice than monovalent cations, Ca ions exhibit sluggish diffusion in inorganic crystals.^[^ ^8^ ^]^ In recent years, a number of inorganic cathode materials are reported for calcium storage, such as Mg_0.25_V_2_O_5_∙H_2_O,^[^ ^7c^ ^]^ K_2_BaFe(CN)_6_,^[^ ^9^ ^]^ Na‐doped NH_4_V_4_O_10_,^[^ ^10^ ^]^ *β*‐Ag_0.33_V_2_O_5_,^[^ ^11^ ^]^ Na*_x_* MnFe(CN)_6_,^[^ ^12^ ^]^ CaCo_2_O_4_,^[^ ^13^ ^]^ VOPO_4_∙2H_2_O,^[^ ^14^ ^]^ NaV_2_(PO_4_)_3_,^[^ ^15^ ^]^ FePO_4_,^[^ ^15^ ^]^ FeV_3_O_9_∙1.2H_2_O,^[^ ^7a^ ^]^ Fe_4_[Fe(CN)_6_]_3_,^[^ ^16^ ^]^ Na_2_FePO_4_F^[^ ^17^ ^]^ and K_0.5_V_2_O_5_.^[^ ^7b^ ^]^ Most of these materials show poor rate performance and cycle life. Therefore, the exploitation of high‐performance electrode capable of reversible Ca^2+^ ion storage is particularly important for the development of CIBs.

Alternatively, organic materials, which compose of inexpensive and sustainable elements such as C, O, H, N, and S, have become a promising candidate for energy storage devices.^[^ ^18^ ^]^ Quinones, a class of carbonyl compounds, possess some intrinsic qualities such as large specific capacity, good electrochemical reversibility, molecular diversity, and structural tailorability.^[^ ^19^ ^]^ In addition, the advantage of enolization redox chemistry and easy electron sharing of quinones contribute to low repulsion for Ca‐ion diffusion in solid hosts.^[^ ^20^ ^]^ Organics usually have pseudocapacitive properties, which benefit the acceleration of reaction kinetics and improve Ca‐ion storage ability. So the fast kinetics property of Ca^2+^ can be obtained by means of organic electrode materials. Compared to small quinone molecules, quinone polymers possess low solubility in the solvent, which contributes to long cycle stability. However, the lack of metal elements renders organic materials poor electronic conductivity, leading to the addition of more conductive additives. At present, many works have studied organic materials in the aqueous Ca‐ion battery, such as 3,4,9,10‐perylene tetracarboxylic dianhydride (PTCDA),^[^ ^21^ ^]^ 5,7,12,14‐pentacenetetrone (PT),^[^ ^22^ ^]^ and polyimide poly[*N*,*N′*‐(ethane‐1,2‐diyl)‐1,4,5,8‐naphthalenetetracarboxiimide] (PNDIE).^[^ ^20d^ ^]^ Despite the promising potential of using organic electrodes for Ca‐ion storage, a few studies in nonaqueous systems have been demonstrated so far.

Here in, we report a quinone polymer, represented by in situ formed poly(anthraquinonyl sulfide)@CNT (PAQS@CNT), as high rate performance CIB cathode material in nonaqueous electrolyte. The introduction of carbon nanotubes (CNT) effectively improved the electrical conductivity of PAQS and reduced voltage gaps between oxidation state and reduction state of PAQS, providing a fast ion diffusion (diffusion coefficient on the order of 10^−6^–10^−9^ cm^2^ S^−1^). PAQS@CNT was used as work electrode, active carbon as counter electrode, and Ag^+^/Ag as reference electrode. All of the electrochemical performance tests used the three‐electrode system. As expected, the PAQS@CNT cathode shows satisfactory Ca‐ion storage ability. The electrode exhibits a specific capacity of 116 mAh g^−1^ at a specific current of 0.05 A g^−1^, a superior rate capacity of 60 mAh g^−1^ at 4 A g^−1^, and a good cycle stability of 83% retention at 1 A g^−1^ after 500 cycles. The mechanism study through ex situ Fourier transform infrared spectroscopy (FTIR) and X‐ray photoelectron spectroscopy (XPS) suggests that Ca^2+^ and Ca(TFSI)^+^ coordinated with C═O of PAQS. Furthermore, binding energy calculations suggest that the construction of Ca^2+^ and Ca(TFSI)^+^ co‐intercalation in the PAQS is the most reasonable. These findings contribute to a better understanding of the charge storage behavior of organic electrode materials in CIBs.

## 2 Results and Discussion

**Figure 1:**
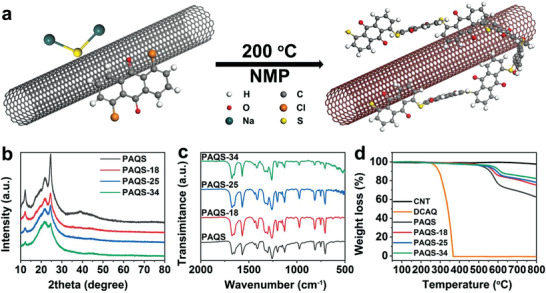
a) In situ polymerization process of PAQS@CNT composite. b) XRD patterns of PAQS, PAQS‐18, PAQS‐25, and PAQS‐34. c) FTIR spectra of PAQS, PAQS‐18, PAQS‐25, and PAQS‐34. d) TGA curves of CNT, DCAQ, PAQS, PAQS‐18, PAQS‐25, and PAQS‐34.

Typical synthetic process of in situ formed PAQS@CNT composites is similar to that of bare PAQ except for additional CNT being added in the raw material with ultrasonic dispersion before polymerization. The preparation process is displayed in **Figure** **1**a (synthetic detail in the “Experimental Section”). To study the effect of CNT content on the electrochemical performance, we prepared different CNT content composites, named bare PAQS (without CNT), PAQS‐18 (PAQS@18%CNT), PAQS‐25 (PAQS@25%CNT), and PAQS‐34 (PAQS@34%CNT). As shown in Figure 1b, the X‐ray diffraction (XRD) of various CNT proportion PAQS all exhibits consistent diffraction peak, which is consistent with previously reported literature.^[^ ^23^ ^]^ As the amount of CNT content increases, the intensity of the 22° diffraction peak is enhanced, which is due to the influence of CNT diffraction peak at the same degree (Figure S1, Supporting Information). FTIR was performed to identify the structure of PAQS. Four samples show uniform peaks in Figure 1c, where all the peaks have no difference from previous works.^[^ ^6^, ^24^ ^]^ The peaks at 1676 and 1569 cm^−1^ correspond to the stretching vibration of C═O and C═C in the aromatic ring, whereas the peak at 1129 and 1413 cm^−1^ can be attributable to ring–sulfur stretching and ring stretching of the sulfur disubstituted aromatic ring, suggesting the formation of a thioether bond between two monomers.^[^ ^25^ ^]^ To further identify monomer and polymer, Thermogravimetric analysis (TGA) was performed in Figure 1d. The TGA test for CNT, 1,5‐dichloranthraquinone (DCAQ) monomer and different CNT proportion PAQS was performed in the range of 25–800 °C in the N_2_ atmosphere. Starting at 224 °C, the TGA curve of DCAQ monomer rapidly lost weight due to poor thermostability. However, that of bare PAQS exhibits superior thermal stability with slow weight loss, and reserves 62.8 wt% of its initial weight after 800 °C. In addition, the weight of CNT remained almost unchanged from beginning to end. As the CNT content increases in the PAQS, the mass loss of composite decreases, which suggests increment of CNT content in the composite. Compared to poor thermostability of DCAQ, the superior thermal stability of PAQS suggests the success of polymerization. The Raman spectrum for CNT and different CNT proportion PAQS is performed in Figure S2 (Supporting Information). The Raman spectrum of CNT shows a high intensity G peak at 1592 cm^−1^, which suggests the high graphitization of CNT. As the CNT content increases in the PAQS, the intensity of peak at 1592 cm^−1^ gradually increases, which proves the incremental CNT content in the composite again. The above results of XRD, FTIR, TGA, and Raman spectra can prove that bare PAQS polymer and PAQS@CNT composites were successfully obtained.

**Figure 2:**
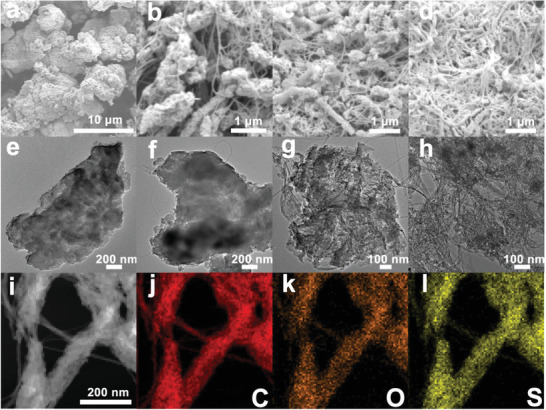
SEM images of a) PAQS, b) PAQS‐18, c) PAQS‐25, and d) PAQS‐34. TEM images of e) PAQS, f) PAQS‐18, g) PAQS‐25, and h) PAQS‐34. i–l) EDS mapping image of PAQS‐34.

To study the morphology change of PAQS composited with various percent proportions of the CNT, scanning electron microscope (SEM), and transmission electron microscope (TEM) were performed. As shown in **Figure** **2**a, the morphology of bare PAQS shows irregular micrometer‐sized bulk. In Figure 2b–d, CNT originally is sparsely attached to the surface of PAQS. However, with further increase in the content of CNT, CNT is closely and uniformly combined with PAQS, which is more beneficial to the enhancement of the conductivity of PAQS. TEM also shows good combination between PAQS and CNT with the addition of the CNT in Figure 2e–h. The element mappings of PAQS‐34 show that the C, O, and S elements are uniformly distributed in the composite, which further proves that PAQS and CNT are homogeneously composited (Figure 2i–l).

**Figure 3:**
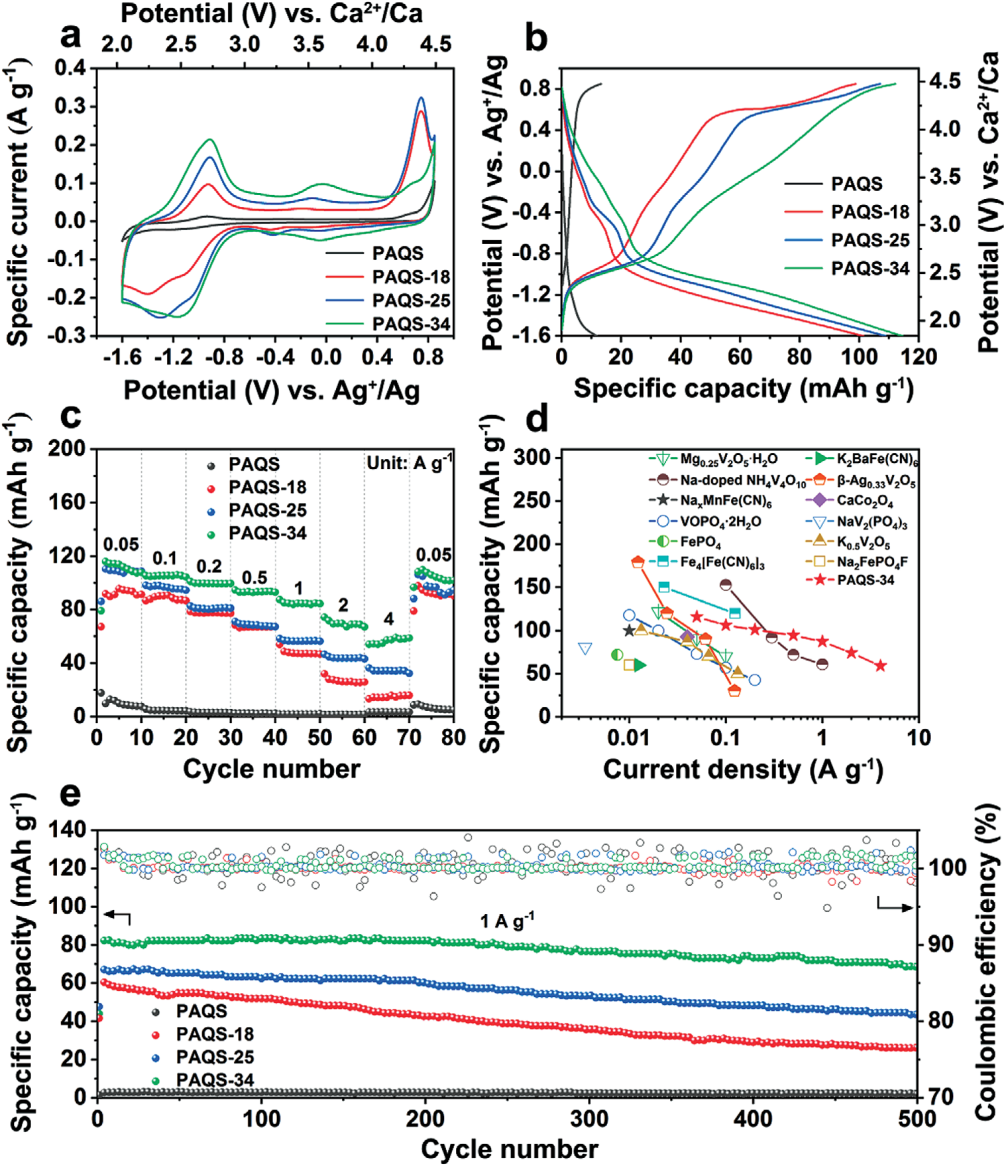
Electrochemical performance of PAQS, PAQS‐18, PAQS‐25, and PAQS‐34. a) CV curves at 0.5 mV s^−1^. The standard potential of Ag^+^/Ag reference electrode was calibrated by ferrocene in Figure S3 (Supporting Information). b) GCD curves at a current density of 0.1 A g^−1^ in the voltage range from −1.6 to 0.85 V versus Ag^+^/Ag. c) Rate performance at different current densities. d) Capacity and rate ability comparison with reported inorganic cathodes for nonaqueous CIBs. See Table S4 (Supporting Information) for reported data. e) Long‐term cycle performance at a current density of 1 A g^−1^ for 500 cycles.

**Figure 4:**
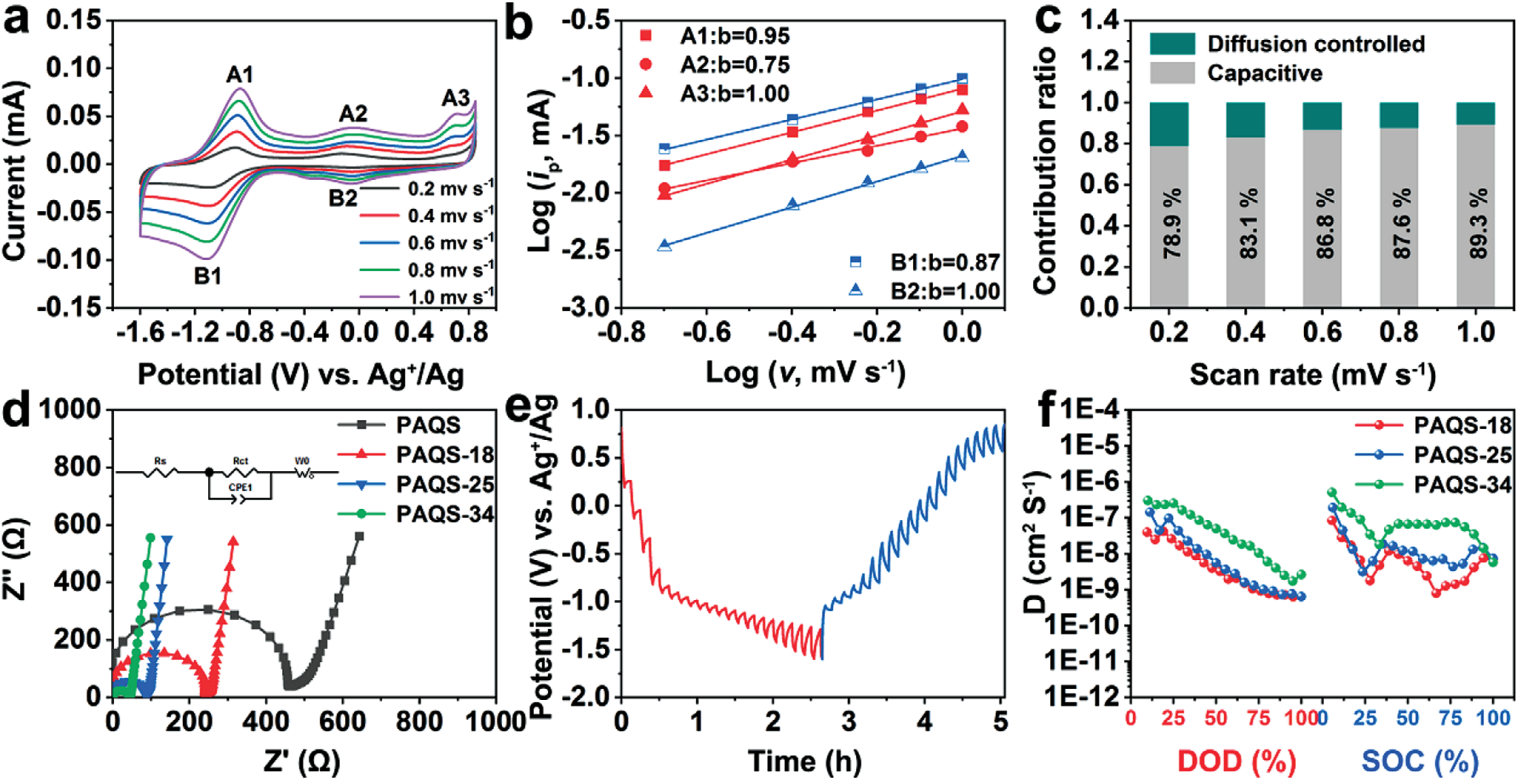
a) CV curves of PAQS‐34 at scanning rates from 0.2 to 1.0 mV s^−1^. b) log (v) versus log (i) plots of PAQS‐34. c) Contribution ratio of the capacitive and diffusion‐controlled processes of PAQS‐34 at various scan rates. d) EIS curves of PAQS, PAQS‐18, PAQS‐25, and PAQS‐34 with applied inset equivalent circuit. e) GITT curves of PAQS‐34 during discharge and charge at a current density of 0.1 A g^−1^. f) Ca^2+^ ion diffusion coefficient during discharging and charging processes in PAQS‐18, PAQS‐25, and PAQS‐34.

The electrochemical performance of diverse percentage CNT content PAQS was tested using a three‐electrode battery. PAQS was used as the working electrode, and active carbon and Ag^+^/Ag were used as counter electrode and reference electrode, respectively. To evaluate the electrochemical performance of different percentage CNT contents of PAQS, cyclic voltammetry (CV) profiles were performed at 0.5 mV s^−1^ in the voltage range from −1.6 to 0.85 V versus Ag^+^/Ag (**Figure** **3**a). The CV curve of bare PAQS shows hardly any electrochemical activity due to its poor electronic conductivity. However, PAQS‐18 shows three obvious reduction peaks at −0.44, −1.1, and −1.4 V versus Ag^+^/Ag and two oxidization peaks at −0.93 and 0.74 V versus Ag^+^/Ag, which can be ascribed to the fact that CNT speeded up the transmission of electrons and diffusion of Ca^2+^. Hence the electrochemical activity of PAQS‐18 was significantly enhanced. It is worth noting that PAQS‐18 exhibits asymmetrical redox peaks, and 1180 mV voltage gaps between −0.44 V (red.) versus Ag^+^/Ag and 0.74 V (ox.) versus Ag^+^/Ag, and 470 mV voltage gaps between −1.4 V (red.) versus Ag^+^/Ag and −0.93 V (ox.) versus Ag^+^/Ag. This asymmetric redox peak behavior has also been observed in several other cathodes for Ca^2+^ storage.^[^ ^7^, ^12^, ^26^ ^]^ However, there is no clear explanation for this phenomenon. Based on the reported literature, the reason might be the intercalation/extraction of Ca^2+^ and Ca(TFSI)^+^ that occurs in stages. The reduction steps occur faster than oxidation steps, so the voltage gap between the two steps is too small to distinguish the steps. In addition, the voltage hysteresis leads to a voltage gap between the redox peaks. Some conjugated carbonyl‐based organic materials and metal fluorides/oxides with multielectron reactions also show the same phenomenon.^[^ ^27^ ^]^ With the increasing CNT content, PAQS‐25 shows higher electrochemical activity and lesser voltage gaps of 380 mV between −1.3 V (red.) versus Ag^+^/Ag and −0.92 V (ox.) versus Ag^+^/Ag. When the CNT content is up to 34%, the oxidation state at 0.74 V versus Ag^+^/Ag becomes weaker. Instead, PAQS‐34 shows two pairs of symmetrical redox peaks at −1.16 V (red.) and −0.92 V (ox.) versus Ag^+^/Ag and at −0.05 V (red.) and −0.03 V (ox.) versus Ag^+^/Ag, which show 240 and 20 mV voltage gaps between reduction state and oxidation state, respectively. Symmetrical redox peaks and less voltage gaps prove that the redox reactions of PAQS‐34 are highly reversible due to enhanced electronic conductivity. The change of the CV curve shape is also related to Ca^2+^ diffusion rate, which will be analyzed later in Figure 4f. The oxidization peak at 0.85 V versus Ag^+^/Ag is attributed to the common capacity contribution of PAQS and CNT (Figure S4, Supporting Information). The CV measurement was also applied to Al foil in the same condition. As shown in Figure S5 (Supporting Information), the current intensity of Al foil is negligible compared with that of PAQS‐34, which suggests that there is no side reaction on Al foil during the electrochemical test. Besides, the linear sweep voltammetry (LSV) of electrolyte is also measured in Figure S6 (Supporting Information). The results show that the electrolyte is stable at 0.85 V versus Ag^+^/Ag.

Figure 3b shows the galvanostatic charge–discharge (GCD) profiles of various percentage CNT content PAQS. The voltage range is also from −1.6 to 0.85 V versus Ag^+^/Ag. Bare PAQS only has 12 mAh g^−1^. But the capacity of PAQS‐18 is significantly enhanced to 101 mAh g^−1^ at 0.1 A g^−1^ after the addition of CNT. In addition, the specific capacity values of PAQS‐25 and PAQS‐34 are further increased to 109 and 115 mAh g^−1^, respectively. Moreover, the potential plateaus in the GCD curves accord with the CV result. The capacity contribution of CNT was also tested at various current densities in Figure S7 (Supporting Information), and the CNT only provides 9, 13, and 18 mAh g^−1^ at 0.1 A g^−1^ in the PAQS‐18, PAQS‐25, and PAQS‐34, respectively. Figure 3c shows the rate performance of four kinds of PAQS at various current densities from 0.05 to 4 A g^−1^. PAQS‐34 exhibits optimal rate capability than others, because improved electronic conductivity contributes to superior kinetic property. The high specific capacity of PAQS‐34 can reach 60 mAh g^−1^ at 4 A g^−1^, which recovers to 109 mAh g^−1^ when the current density is changed to 0.05 A g^−1^. The GCD curves of PAQS‐34 at different current densities are shown in Figure S8 (Supporting Information). The demonstrated rate performance surpasses currently reported inorganic cathode materials for nonaqueous CIBs (Figure 3d; Table S4, Supporting Information). The current density of majority of reported cathode materials can only operate below 0.1 A g^−1^ due to strong interaction between Ca^2+^ and hosting lattice in inorganic materials. However, enolization redox chemistry of organics accelerates Ca^2+^ ion diffusion with low repulsion, which leads to the exhibition of superior rate performance of PAQS‐34. More importantly, organic materials usually undergo dissolution in the organic solvent, causing capacity fading. Nevertheless, the composite of PAQS and CNT inhibits the dissolution to some extent. Through membrane color change after 500 cycles at a current density of 1 A g^−1^, the light‐colored membrane suggests the lower solubility of PAQS‐34 with the increasing CNT content (Figure S9, Supporting Information). The long‐term cycle stability was performed at a current density of 1 A g^−1^ in Figure 3e. PAQS‐34 exhibits outstanding cycling performance. After 500 cycles, a discharge capacity of 68.6 mAh g^−1^ is sustained, retaining 83% of initial capacity. Conversely, PAQS‐18 and PAQS‐25 only show 42% and 65% capacity retention ratios due to material dissolution. In addition, the Coulombic efficiency of PAQS‐34 is nearly 100%, which proves the high electrochemical reversibility in the PAQS‐34. Besides, PAQS‐18, PAQS‐25, and PAQS‐34 show no capacity decay at a current density of 0.5 A g^−1^ for 100 cycles (Figure S10, Supporting Information). To further study the effect of higher CNT content, PAQS‐41 (PAQS@41%CNT) was prepared. PAQS‐41 shows poor uniformity due to an excess of CNT (Figure S12, Supporting Information). By comparing electrochemical performance, PAQS‐41 shows inferior capacity and rate performance than PAQS‐34 (Figures S13–15, Supporting Information).

To explore the mechanism of superior rate performance of PAQS‐34, the electrochemical reaction kinetics were studied by recording the CV curves at different scan rates. **Figure** **4**a shows the CV curves of PAQS‐34 at scan rates of 0.2–1.0 mV s^−1^, and the curves display consistent shapes with cumulative specific currents. To compare the reaction kinetics of PAQS‐18, PAQS‐25, and PAQS‐34, oxidation peaks were chosen, which were defined as A1, A2, and A3, and reduction peaks defined as B1 and B2. In principle, the measured current (i) can be correlated to the scanning rate (v) on the basis of Equation (3) (detailed introduction in the “Experimental Section”).^[^ ^28^ ^]^ If the *b*‐value is 0.5, the contribution of capacity is from the diffusion‐controlled behavior. In contrast, if the *b*‐value is 1, it represents an ideal capacitive process. As shown in Figure 4b, the *b*‐values of redox peaks are all close to 1, suggesting that the main contribution of capacity is from capacitive behavior. The *b*‐values of PAQS‐18 and PAQS‐25 are also shown in Figures S16b and S17b (Supporting Information), respectively, which exhibit similar capacitive dominated result. To further quantify the proportion of capacitive and diffusion‐controlled contributions, the capacitive contribution at various scan rates is analyzed via Equation (4) (detailed introduction in the “Experimental Section”).^[^ ^29^ ^]^ Figure 4c exhibits capacitive contribution of PAQS‐34 at various scan rates. The contribution of capacitive increases with the increase of scan rates. Figure S18 (Supporting Information) shows that 89.3% of the total capacity originates from capacitive contribution at a scan rate of 1.0 mV s^−1^, which is beneficial to high rate performance and long cycle stability. The capacitive contribution of PAQS‐18 and PAQS‐25 also shows the same growth trend with increased scan rates in Figures S16c and S17c (Supporting Information), respectively. By comparison, the value of capacitive contribution of PAQS‐34 is all higher than PAQS‐18 and PAQS‐25 at different scan rates, which corresponds to higher rate performance in PAQS‐34. Electrochemical impedance spectroscopy (EIS) was performed to study the charge‐transfer kinetics of different CNT percentages of PAQS. As shown in Figure 4d, two regions can be observed, including one semicircle at high frequency and a slope line at low frequency, which correspond to charge‐transfer and diffusion processes. The equivalent circuit is shown in Figure 4d (inset). The charge‐transfer resistance values (*R*_ct_) of PAQS, PAQS‐18, PAQS‐25, and PAQS‐34 are fitted to about 421, 231, 78, and 38 Ω, respectively. The lowest charge‐transfer resistance of PAQS‐34 is beneficial to fast ion transport and the charge‐transfer process. However, the charge‐transfer resistance (*R*_ct_) of PAQS‐41 is fitted to about 42 Ω (Figure S19, Supporting Information). Since excess CNT will be hard to disperse leading to agglomeration, the dispersity of PAQS in CNT becomes worse. The conductive network of PAQS‐41 composite is incomplete, so PAQS‐41 shows larger impedance than PAQS‐34. The diffusion of Ca^2+^ ions in different CNT percentage PAQS was quantified by galvanostatic intermittence titration technique (GITT). The electrode undergoes a series of current pluses for 150 s and is followed by a relaxation process for 300 s to reach equilibrium (Figure 4e; Figure S20, Supporting Information). The ionic diffusion coefficient (*D*) of Ca^2+^ in the active material can be determined by Equation (5) (detailed introduction in the “Experimental Section”).^[^ ^30^ ^]^ The calculated diffusion coefficient of the Ca^2+^ ions (*D*) in the PAQS‐34 during discharging and charging is in the range of 10^−6^–10^−9^ cm^2^ S^−1^ (Figure 4f), which is higher than PAQS‐18 and PAQS‐25. The high diffusion coefficient of Ca^2+^ leads to improved reaction kinetics of PAQS during the electrochemical reaction process. Interestingly, the diffusion coefficient of intermediate state of charge (SOC) is lower than high SOC during the charge process in the PAQS‐18. In contrast, the diffusion coefficient of intermediate SOC is higher than high SOC in the PAQS‐34, which suggests that the change of Ca^2+^ diffusion coefficient is consistent with the change of the CV curve shape. Due to enhanced Ca^2+^ diffusion rate, the voltage gaps between reduction state and oxidation state are reduced.

**Figure 5:**
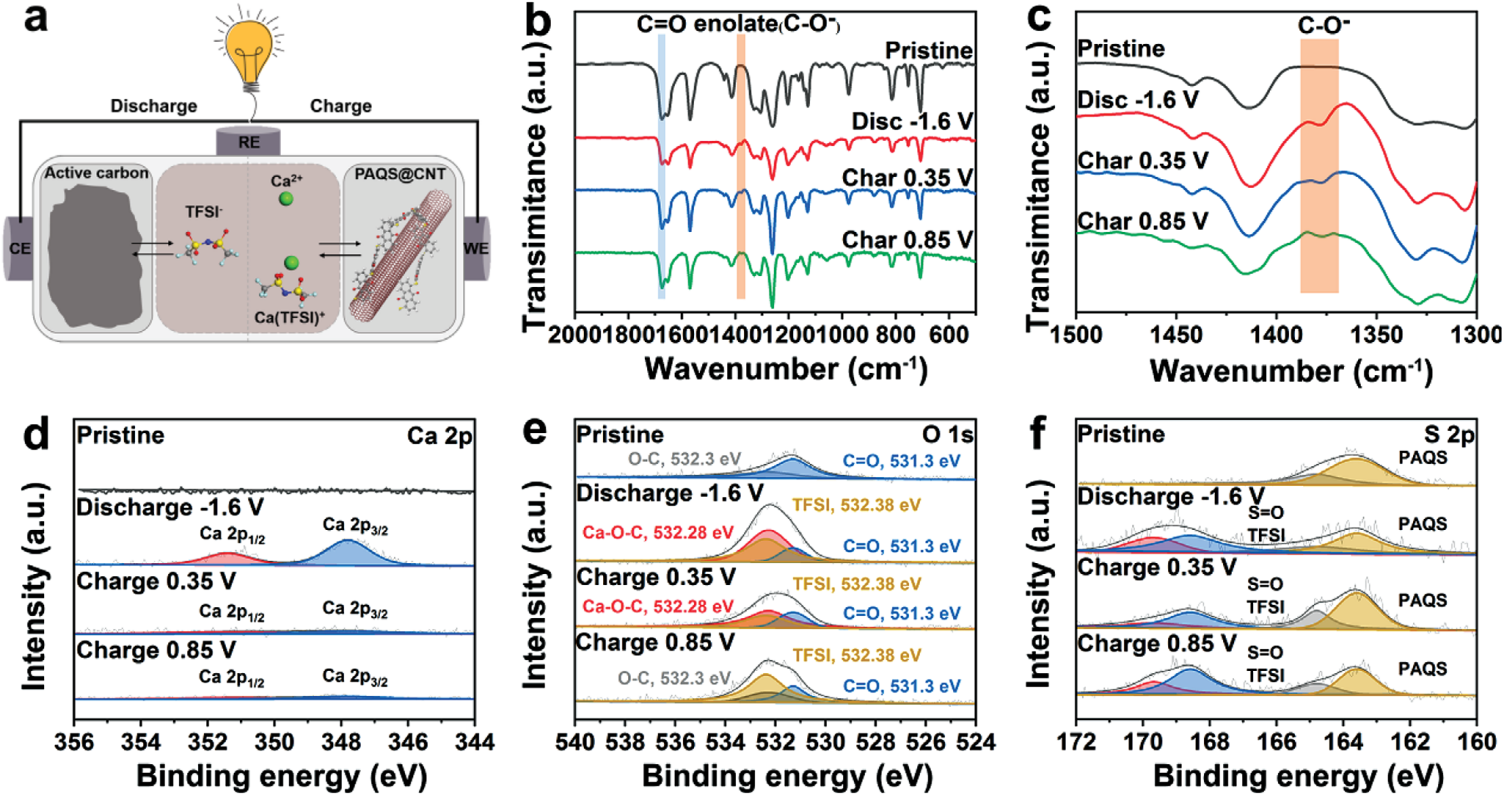
a) Schematic illustration of reaction mechanism. b,c) Ex situ FTIR of PAQS‐34. Ex situ XPS spectra of PAQS‐34 d) Ca 2p, e) O 1s, and f) S 2p.

The configuration and reaction mechanism of CIB are shown in **Figure** **5**a. On discharging, Ca^2+^ and Ca(TFSI)^+^ co‐intercalate in the PAQS and combine with their carbonyl bonds. Meanwhile, active carbon adsorbs TFSI^−^ anions in the electrolyte. In the charge process, Ca^2+^ and Ca(TFSI)^+^ de‐intercalate from the PAQS, and TFSI^−^ desorbs from the active carbon and diffuses back into the electrolyte. To further prove our theory of reaction mechanism, ex situ FTIR and ex situ XPS were performed with the PAQS‐34 at the first cycle. FTIR spectra of PAQS‐34 at different voltage states are shown in Figure 5b,c. In the FTIR spectra, upon discharging, the intensity of carbonyl group (C═O) at 1676 cm^−1^ is reduced. In addition, a new band at 1374 cm^−1^ is observed, which suggests the conversion of carbonyl group (C═O) to enolate group (C—O^−^).^[^ ^6^, ^31^ ^]^ After the charging process, the intensity of the carbonyl group (C═O) recovers, and the peak of the enolate group gradually disappears, indicating the reversibility of the conversion process. In addition, XPS Ca 2p spectra reveal the reversible uptake of Ca^2+^ during discharge and de‐intercalate after charging (Figure 5d). It is worth noting that Ca^2+^ is almost totally removed from PAQS‐34 during the charging process at 0.35 V versus Ag^+^/Ag. The reduction of voltage gaps makes most of capacity contribution during 0.35–0.85 V versus Ag^+^/Ag transfers to the voltage range below 0.35 V versus Ag^+^/Ag. In contrast, XPS Ca 2p spectra of PAQS‐18 and PAQS‐25 demonstrate obvious removal of Ca^2+^ from PAQS during the charging process at the voltage of 0.35–0.85 V versus Ag^+^/Ag (Figures S21 and S22, Supporting Information). In addition, XPS O 1s spectra show the reversible intensity change of C═O/Ca—O—C,^[^ ^32^ ^]^ which is consistent with the FTIR spectra result (Figure 5e). It is worth noting that O 1s at 532.38 eV from TFSI^−^ anion (Figure S23, Supporting Information) shows the change of intensity, which is related to the intercalation/extraction of Ca(TFSI)^+^ in the PAQS and TFSI^−^ in the CNT. Detailed explanations will be given with the aid of XPS S 2p and F 1s spectra. The XPS S 2p spectra of PAQS‐34 are shown in Figure 5f. The pristine state of PAQS‐34 only shows S 2p_1/2_ and S 2p_3/2_ at 164.8 and 163.6 eV from PAQS. However, the new peak of S═O from TFSI^−^ anion appears at 169.6 and 168.5 eV at the discharged state, which suggests the co‐insertion of Ca(TFSI)^+^ in the discharging process.^[^ ^33^ ^]^ The XPS S 2p spectra of Ca(TFSI)_2_ powder are shown in Figure S24 (Supporting Information), which prove that the new peak is S═O from TFSI^−^ anion. After being charged to 0.35 V versus Ag^+^/Ag, the peak intensity of TFSI^−^ was decreased because Ca(TFSI)^+^ was removed from PAQS‐34 during the charging process. When charged to 0.85 V versus Ag^+^/Ag, the peak intensity of TFSI^−^ was increased again, which is due to the intercalation of TFSI^−^ into CNT during high potential.^[^ ^34^ ^]^ The ex situ XPS F 1s spectra of PAQS‐34 also show the same tendency in Figure S25 (Supporting Information). So it is difficult to determine whether there is Ca(TFSI)^+^ extraction during the charging process at the voltage of 0.35–0.85 V versus Ag^+^/Ag. The XPS S 2p and F 1s spectra of CNT are also performed in Figures S27 and S28 (Supporting Information). Similarly, the peak intensity of TFSI^−^ was enhanced during the potential charged from 0.35 to 0.85 V versus Ag^+^/Ag, which further proves the intercalation of TFSI^−^ in CNT. The result of the atomic content of the elements for PAQS‐34 is demonstrated in Table S3 (Supporting Information). The Ca content is ≈6 times higher in the discharged than the charged state, which corresponds to the increased intensity of the O 1s peak assigned to Ca—O—C. The content of active N was increased in the discharged state due to the intercalation of Ca(TFSI)^+^ in PAQS‐34. In addition, the content of active N was decreased during the charging process at the voltage from −1.6 to 0.35 V versus Ag^+^/Ag, and increased during the charging process at the voltage of 0.35–0.85 V versus Ag^+^/Ag, which corresponds to the de‐intercalation of Ca(TFSI)^+^ from PAQS‐34 and the intercalation of TFSI^−^ anion into CNT. The Ca/N_active_ ratio in the discharged state is found to be 2. Because the fact that the Ca/N ratio of Ca(TFSI)^+^ is 1, the Ca^2+^/Ca(TFSI)^+^ ratio of intercalation is ≈1, which confirms the co‐intercalation of Ca^2+^ and Ca(TFSI)^+^ in the PAQS.

To further prove that the construction of Ca^2+^ and Ca(TFSI)^+^ co‐intercalation is reasonable, we conducted the computational simulation for binding energies of PAQS using the density function theory with Dmol3 module in Materials Studio.^[^ ^35^ ^]^ The details of computational simulation are provided in the Supporting Information. The dissociation of Ca^2+^ from electrolyte and its combination with PAQS involve two steps, which conform to the following equations

(1) $$\mathrm{Ca}{\left(\mathrm{TFSI}\right)}_{2}\;+\;\mathrm{PAQS}\;+\;\mathrm{AC}>\mathrm{PAQS}\;-\;\mathrm{Ca}{\left(\mathrm{TFSI}\right)}^{+}\;+\;\mathrm{AC}\;-\;\mathrm{TFS}I^{-}$$

(2) $$\mathrm{PAQS}-\mathrm{Ca}{\left(\mathrm{TFSI}\right)}^{+}+\mathrm{AC}>\mathrm{PAQ}-{\mathrm{Ca}}^{2+}+\mathrm{AC}-\mathrm{TFS}I^{-}$$

**Figure 6:**
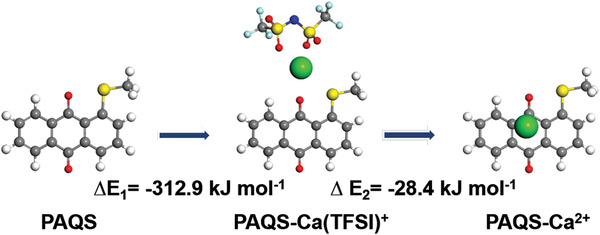
Optimized geometries of the bare PAQS, PAQS–Ca(TFSI)^+^, and PAQS–Ca^2+^.

Optimized geometries of the bare PAQS, PAQS‐Ca(TFSI)^+^, and PAQS‐Ca^2+^ are demonstrated in **Figure** **6**. The binding energies of the two steps are ∆*E*_1_ = −312.9 kJ mol^−1^ and ∆*E*_2_ = −28.4 kJ mol^−1^, respectively. It is to be observed that more negative binding energy means the favorable binding environment of PAQS–Ca(TFSI)^+^. Although ∆*E*_2_ is higher than ∆*E*_1_, the equilibrium of the second step (Equation (2)) is still moving to the right because the binding energy is negative. Therefore, the co‐existence of PAQ–Ca^2+^ and PAQS–Ca(TFSI)^+^ in the PAQS is the most reasonable result.

## 3 Conclusions

In sum, the strong electrostatic interaction between Ca^2+^ and hosting lattice and the large ion radius of Ca^2+^ (1.0 Å) lead to poor kinetics performance. Sluggish Ca^2+^ diffusion makes the fast storage of Ca^2+^ highly challenging. We proposed an in situ formed PAQS@CNT composite as a high‐rate‐performance CIB cathode. Benefited from the fast reaction mechanism of organics and improved electronic conductivity by the introduction of CNT, the diffusion coefficient of Ca^2+^ can be improved to 10^−6^–10^−9^ cm^2^ S^−1^ by the GITT technique. The PAQS‐34 cathode displayed a specific capacity of 116 mAh g^−1^ at 0.05 A g^−1^ and 60 mAh g^−1^ at 4 A g^−1^, which exhibits outstanding rate performance and exceeds most inorganic cathode materials in nonaqueous CIBs. The introduction of CNT not only reduced the voltage gaps between oxidation state and reduction state of PAQS but also improved the cycling stability by inhibiting material dissolution. Ex situ FTIR and XPS collectively proved reversible Ca^2+^ and Ca(TFSI)^+^ storage chemistry. Computational simulations suggest that Ca^2+^ and Ca(TFSI)^+^ prefer to co‐intercalate in the PAQS. Because investigations of CIBs are still in the early stage, this work provides a new avenue for high‐rate‐performance cathode material for rechargeable CIBs. However, excessive CNT added in the electrode will lead to a decrease in volume energy density. Therefore, only by developing organic materials with high electronic conductivity and reducing the amount of conductive additives, and on the premise of not reducing the volume energy density of electrode, we can make better use of pseudocapacitance properties to construct high‐performance electrode materials for calcium‐ion battery.

## 4 Experimental Section

### Synthesis of PAQS and PAQS@CNT Composites

PAQS@CNT composite was synthesized by a typical method.^[^ ^36^ ^]^ DCAQ (0.554 g, 0.002 mol) and single‐wall CNTs (SWCNTs) (0.0831, 0.1385, 0.1939, and 0.2493 g) were first mixed in 10 mL *N*‐methylpyrrolidone (NMP). The mixture was then sonicated for 20 min and transferred into a flask. After adding sodium sulfide nonahydrate (0.48 g, 0.002 mol) into the mixture, the suspension was stirred under Ar at 200 °C for 12 h. The product was centrifuged after cooling to room temperature, and followed by 2 days Soxhlet extracted with water and acetone, respectively. The finally product was dried at 120 °C for 12 h to obtain PAQS‐18, PAQS‐25, PAQS‐34, and PAQS‐41. The bare PAQS was also synthesized through the same process without adding SWCNTs. The molecular formula of PAQS is Cl(C_14_H_6_O_2_S)*_n_* Cl. The synthetic structure diagram of PAQS is shown in Figure S30 (Supporting Information). The matrix‐assisted laser desorption/ionization time‐of‐flight (MALDI‐TOF) spectrum showed oligomers with up to 17 repeating units (Figure S31, Supporting Information).

### Physical Characterization

The crystal structures of the materials were studied by XRD (Brucker D8 X‐ray diffractometer) with Cu K*α* radiation (*λ* = 1.5418) at room temperature. IR spectra were measured with KBr pellets on a Bruker IFS‐66 V/S FTIR spectrometer. Thermogravimetric measurement was performed on preweighed samples in a nitrogen stream using a Netzsch STA 449C apparatus with a heating rate of 10 °C min^−1^ under N_2_ atmosphere. The morphologies of the materials were investigated using a field emission SEM (JEOL JSM‐6700F) and TEM (FEI Tecnai G2 F20 S‐TWIN). XPS was performed on ESCALAB 250 with Mg K*α* as the X‐ray source. The Raman spectroscopy was tested using a Renishaw in via Raman microscope with Ar‐ion laser excitation (*λ* = 514.5 nm). MALDI‐TOF mass spectroscopy was recorded on a Bruker Autoflex II spectrometer. PAQS was measured in positive ion mode using trans‐2‐[3‐(4‐tert‐butylphenyl)‐2‐methyl‐2‐propenylidene]malononitrile (DCTB) as the matrix.

### Electrochemical Measurement

The working electrodes (PAQS, PAQS‐18, PAQS‐25, PAQS‐34, and PAQS‐41) were prepared by mixing the active materials, Super P, and polyvinylidene fluoride (PVDF) binders in weight ratios of 7:2:1 with appropriate amount of NMP to form a homogeneous slurry. The slurry was then coated on an aluminum foil current collector (thickness of 20 µm) and transferred to a vacuum oven at 120 °C for 10 h. The electrodes were divided into a disk of 5 mm diameter, and the glass fiber filters (Waterman GF/C) were divided into a disk of 6 mm diameter. The electrolyte was composed of 0.8 m Ca(TFSI)_2_ dissolved in a mixture of ethylene carbonate (EC), dimethyl carbonate (DMC), propylene carbonate (PC), ethylmethyl carbonate (EMC) (vol/vol/vol/vol = 2:3:2:3). The loading mass of the working electrode was around 1.0 mg cm^−2^. The counter electrode was prepared by mixing active carbon (XFNANO, Inc.) and polytetrafluoroethylene (PTFE) in a weight ratio of 9:1 with ethyl alcohol. After natural evaporation, the mixture was flatted and then dried in the oven at 60 °C for 1 h. The counter electrode was also divided into a disk of 5 mm diameter, and an excessive mass loading of 3.3 mg cm^−2^ was employed. As for the density of the electrode, the PAQS, PAQS‐18, PAQS‐25, and PAQS‐34 showed 0.60, 0.54, 0.45, and 0.38 g cm^−3^ respectively. The porosity of the PAQS, PAQS‐18, PAQS‐25, and PAQS‐34 showed 10%, 28%, 40%, and 50% respectively.

For the test of electrochemical performance of PAQS, a three‐electrode battery (Swagelok) was assembled using a PAQS working electrode, an active carbon counter electrode, and a nonaqueous Ag^+^/Ag reference electrode. SUS (316) disk electrode was used as a conductor. The structure diagram of the three‐electrode battery (Swagelok) is shown in Figure S32 (Supporting Information). Three‐electrode batteries were assembled in an argon‐filled glove box. Galvanostatic charge–discharge cycling was tested on a land‐2001A (Wuhan, China) automatic battery tester. CV and EIS were tested on a CHI660E electrochemical workstation (Shanghai Chenhua, China). All electrochemical tests were conducted in an argon‐filled glove box at room temperature.

In principle, the measured current (i) can be correlated to the scanning rate (v) on the basis of Equation (3)^[^ ^28^ ^]^

(3) $$i\;=\;av^{b}$$

where *a* and *b* are constants. The *b*‐value can be determined by the slope of the log (v)–log (i) plots.

To further quantify the proportion of capacitive and diffusion‐controlled contributions, the capacitive contribution at various scan rates was analyzed via Equation (4)^[^ ^29^ ^]^

(4) $$i_{\left(V\right)}=k_{1\left(V\right)}\;v\;+\;k_{2\left(V\right)}v^{1/2}$$

where *k*_1(_ *_V_*_)_ *v* and *k*_2(_ *_V_*_)_ *v* ^1/2^ represent capacitive and diffusion‐controlled contributions, respectively. The values of *k*_1(_ *_V_*_)_ and *k*_2(_ *_V_*_)_ can be determined from the slope and the *y*‐axis intercept point for *i*_(_ *_V_*_)_/*v* ^1/2^ as a linear function of *v* ^1/2^ at each fixed potential.

The diffusion of Ca^2+^ ions in different CNT percentage PAQS was quantified by GITT. The electrode was undergone a series of current pluses for 150 s and was followed by a relaxation process for 300 s to reach equilibrium (Figure 4e; Figure S20, Supporting Information). The ionic diffusion coefficient (*D*) of Ca^2+^ in the active material can be determined by Equation (5)^[^ ^30^ ^]^

(5) $$D\;=\frac{4L^{2}}{\pi \tau }\;{\left(\frac{\Delta E_{s}}{\Delta E_{t}}\right)}^{2}$$

where *t* and *τ* refer to the duration time of current pulse (s) and relaxation (s), respectively. *L* represents the Ca^2+^ diffusion distance. Δ*E*_s_ and Δ*E*_t_ correspond to the steady‐state voltage change (*V*) and the voltage change caused by the current pulse, respectively.

## Conflict of Interest

The authors declare no conflict of interest.

## Supporting information

Supporting Information

## Data Availability

Research data are not shared.

## References

[1] a) M. Wang , C. Jiang , S. Zhang , X. Song , Y. Tang , H.‐M. Cheng , Nat. Chem. 2018, 10, 667; 29686378 10.1038/s41557-018-0045-4

[2] C. Lee , S.‐K. Jeong , Chem. Lett. 2016, 45, 1447.

[3] R. J. Gummow , G. Vamvounis , M. B. Kannan , Y. He , Adv. Mater. 2018, 30, 1801702. 10.1002/adma.201801702 29984434

[4] a) A. Ponrouch , C. Frontera , F. Bardé , M. R. Palacín , Nat. Mater. 2016, 15, 169; 26501412 10.1038/nmat4462

[5] a) D. Aurbach , R. Skaletsky , Y. Gofer , J. Electrochem. Soc. 1991, 138, 3536;

[6] a) J. Bitenc , A. Scafuri , K. Pirnat , M. Lozinšek , I. Jerman , J. Grdadolnik , B. Fraisse , R. Berthelot , L. Stievano , R. Dominko , Batteries Supercaps 2020, 4, 214;

[7] a) M. S. Chae , D. Setiawan , H. J. Kim , S.‐T. Hong , Batteries 2021, 7, 54;

[8] T. Chen , G. Sai Gautam , P. Canepa , Chem. Mater. 2019, 31, 8087. 10.3389/fchem.2019.00024 PMC6363690 30761292

[9] P. Padigi , G. Goncher , D. Evans , R. Solanki , J. Power Sources 2015, 273, 460.

[10] T. N. Vo , H. Kim , J. Hur , W. Choi , I. T. Kim , J. Mater. Chem. A 2018, 6, 22645.

[11] J. Hyoung , J. W. Heo , B. Jeon , S.‐T. Hong , J. Mater. Chem. A 2021, 9, 20776.

[12] A. L. Lipson , B. Pan , S. H. Lapidus , C. Liao , J. T. Vaughey , B. J. Ingram , Chem. Mater. 2015, 27, 8442.

[13] M. Cabello , F. Nacimiento , J. R. González , G. Ortiz , R. Alcántara , P. Lavela , C. Pérez‐Vicente , J. L. Tirado , Electrochem. Commun. 2016, 67, 59.

[14] J. Wang , S. Tan , F. Xiong , R. Yu , P. Wu , L. Cui , Q. An , Chem. Commun. 2020, 56, 3805. 10.1039/d0cc00772b 32129434

[15] S. Kim , L. Yin , M. H. Lee , P. Parajuli , L. Blanc , T. T. Fister , H. Park , B. J. Kwon , B. J. Ingram , P. Zapol , R. F. Klie , K. Kang , L. F. Nazar , S. H. Lapidus , J. T. Vaughey , ACS Energy Lett. 2020, 5, 3203.

[16] N. Kuperman , P. Padigi , G. Goncher , D. Evans , J. Thiebes , R. Solanki , J. Power Sources 2017, 342, 414.

[17] A. L. Lipson , S. Kim , B. Pan , C. Liao , T. T. Fister , B. J. Ingram , J. Power Sources 2017, 369, 133.

[18] Y. Liang , Y. Jing , S. Gheytani , K.‐Y. Lee , P. Liu , A. Facchetti , Y. Yao , Nat. Mater. 2017, 16, 841. 28628121 10.1038/nmat4919

[19] C. Han , H. Li , R. Shi , T. Zhang , J. Tong , J. Li , B. Li , J. Mater. Chem. A 2019, 7, 23378.

[20] a) J. Bitenc , A. Scafuri , K. Pirnat , M. Lozinšek , I. Jerman , J. Grdadolnik , B. Fraisse , R. Berthelot , L. Stievano , R. Dominko , Batteries Supercaps 2021, 4, 214;

[21] M. S. Chae , A. Nimkar , N. Shpigel , Y. Gofer , D. Aurbach , ACS Energy Lett. 2021, 6, 2659.

[22] C. Han , H. Li , Y. Li , J. Zhu , C. Zhi , Nat. Commun. 2021, 12, 2400. 33893314 10.1038/s41467-021-22698-9 PMC8065044

[23] a) A. Ahmad , A. Imani , L. Mao , R. Iqbal , H. Zhang , Z. A. Ghazi , R. Ahmad , A. A. Khan , L. Xie , C. M. Chen , Z. Zhang , Z. Wei , Adv. Mater. Technol. 2019, 4, 1900617;

[24] a) Z. Song , H. Zhan , Y. Zhou , Chem. Commun. 2009, 448; 10.1039/b814515f 19137181

[25] a) I. Gomez , O. Leonet , J. Alberto Blazquez , H.‐J. Grande , D. Mecerreyes , ACS Macro Lett. 2018, 7, 419; 10.1021/acsmacrolett.8b00154 35619336

[26] a) T. Tojo , H. Tawa , N. Oshida , R. Inada , Y. Sakurai , J. Electroanal. Chem. 2018, 825, 51;

[27] a) L. Li , R. Jacobs , P. Gao , L. Gan , F. Wang , D. Morgan , S. Jin , J. Am. Chem. Soc. 2016, 138, 2838; 26847657 10.1021/jacs.6b00061

[28] J. Wang , J. Polleux , J. Lim , B. Dunn , J. Phys. Chem. C 2007, 111, 14925.

[29] Z. Wei , X. Meng , Y. Yao , Q. Liu , C. Wang , Y. Wei , F. Du , G. Chen , ACS Appl. Mater. Interfaces 2016, 8, 35336. 27966853 10.1021/acsami.6b12650

[30] D. Chen , M. Lu , B. Wang , R. Chai , L. Li , D. Cai , H. Yang , B. Liu , Y. Zhang , W. Han , Energy Storage Mater. 2021, 35, 679.

[31] a) A. Vizintin , J. Bitenc , A. Kopač Lautar , K. Pirnat , J. Grdadolnik , J. Stare , A. Randon‐Vitanova , R. Dominko , Nat. Commun. 2018, 9, 661; 29445156 10.1038/s41467-018-03114-1 PMC5812995

[32] a) N. Nan , Y. Zhu , Y. Han , Miner. Eng. 2019, 132, 162;

[33] Z. Hawash , L. K. Ono , Y. Qi , Adv. Mater. Interfaces 2016, 3, 1600117.

[34] Z. Chen , T. Liu , Z. Zhao , Z. Zhang , X. Han , P. Han , J. Li , J. Wang , J. Li , S. Huang , X. Zhou , J. Zhao , G. Cui , J. Power Sources 2020, 457, 227994.

[35] a) B. Delley , J. Chem. Phys. 1990, 92, 508;

[36] Z. Song , T. Xu , M. L. Gordin , Y.‐B. Jiang , I.‐T. Bae , Q. Xiao , H. Zhan , J. Liu , D. Wang , Nano Lett. 2012, 12, 2205. 22449138 10.1021/nl2039666

