# Supplementary material for: Poly(Anthraquinonyl Sulfide)/CNT Composites as High‐Rate‐Performance Cathodes for Nonaqueous Rechargeable Calcium‐Ion Batteries
Source: Adv Sci (Weinh). 2022 Mar 20;9(14):2200397. doi: 10.1002/advs.202200397 (PMC9108664; doi:10.1002/advs.202200397)
Supplement: Supplementary file 1 — Supporting Information [file ADVS-9-2200397-s001.pdf]

Supporting Information

Poly(anthraquinonyl sulfide)/CNT Composites as High-Rate-Performance Cathodes for Non-aqueous Rechargeable Calcium-ion Batteries

Siqi Zhang,[a] Youliang Zhu,[b] Denghu Wang,[a] Chunguang Li,[a] Yu Han,[c] Zhan Shi[a] and Shouhua Feng[a]*

[a] S.Q. Zhang, D.H. Wang, C.G. Li, Prof. Dr. Z. Shi, Prof. Dr. S.H. Feng.
State Key Laboratory of Inorganic Synthesis and Preparative Chemistry Jilin University Changchun 130012, P. R. China.
E-mail: zshi@mail.jlu.edu.cn

[b] Prof. Dr. Y. L. Zhu.
State Key Laboratory of Supramolecular Structure and Materials, College of Chemistry, Jilin University, Changchun 130012, P. R. China.

[c] Prof. Dr. Y. Han.
Advanced Membranes and Porous Materials Center, Physical Sciences and Engineering Division, King Abdullah University of Science and Technology (KAUST), Thuwal 23955-6900, Saudi Arabia.

Computation details

All the computations were performed by Density Function Theory with the Dmol3 module of Materials Studio software.[1] In order to calculate geometry optimization and binding energies, we used the generalized gradient approximation (GGA) with the functional proposed by Perdew and Wang (PW91).[2] The binding energies for the two reaction steps were computed as:

$$BE_1 = \left(E_{\mathrm{PAQS-Ca(TFSI)^{+}}} + E_{\mathrm{AC-TFSI^{-}}}\right) - \left(E_{\mathrm{Ca(TFSI)_2}} + E_{PAQS} + E_{AC}\right)$$

$$BE_2 = \left(E_{\mathrm{PAQS-}\quad{}^{2+}} + E_{\mathrm{AC-TFSI^{-}}}\right) - \left(E_{PAQS-Ca(TFSI)^{+}} + E_{AC}\right)$$

where $E_{\mathrm{PAQ-Ca2+}}$, $E_{\mathrm{PAQS-CaTFSI+}}$ and $E_{\mathrm{AC-TFSI-}}$ depict the total energy of the Ca^{2+} and $CaTFSI^{+}$ bind PAQS system and $TFSI^{-}$ bind AC, while the E_{PAQS}, $E_{\mathrm{Ca(TFSI)2}}$ and E_{AC} show the total energies of individual species.

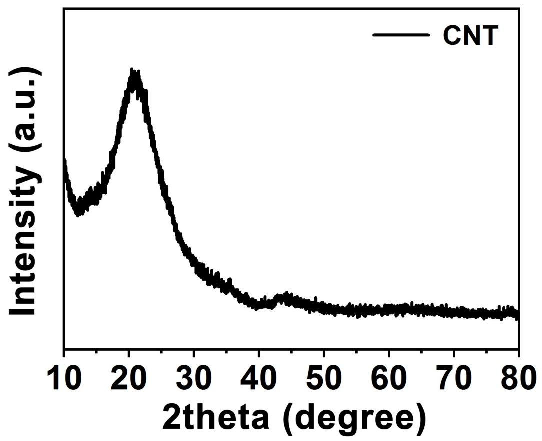

Figure S1. XRD patterns of CNT.

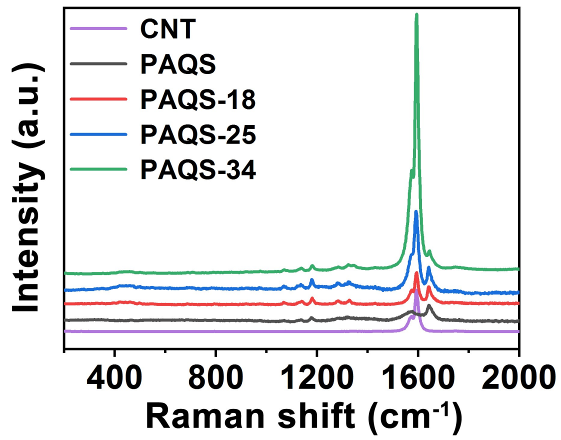

Figure S2. Raman spectrum of CNT, PAQS, PAQS-18, PAQS-25 and PAQS-34.

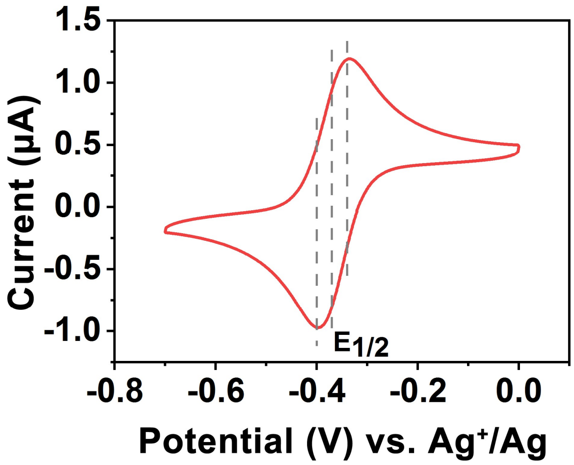

Figure S3. CV curve of 10 mM ferrocene dissolved in 0.8 M $Ca(TFSI)_2$ in EC:DMC:PC:EMC=2:3:2:3 Vol% with Al foil as a working electrode, AC and Ag^+/Ag (0.01 M $AgNO_3$) as counter and reference electrode, respectively. The scan rate is 0.5 mV s^{-1}.

Because the standard potential of Ag^+/Ag reference electrode is unknown, the redox potential of ferrocene was used to calibrate it. The redox potential of ferrocene is -0.37 V vs. Ag^+/Ag, and the potential of reported literature ferrocene against standard hydrogen electrode is 0.4 V.[3] Hence, the $Ag^+/$ Ag reference potential is 3.64 V vs. Ca^{2+}/Ca.

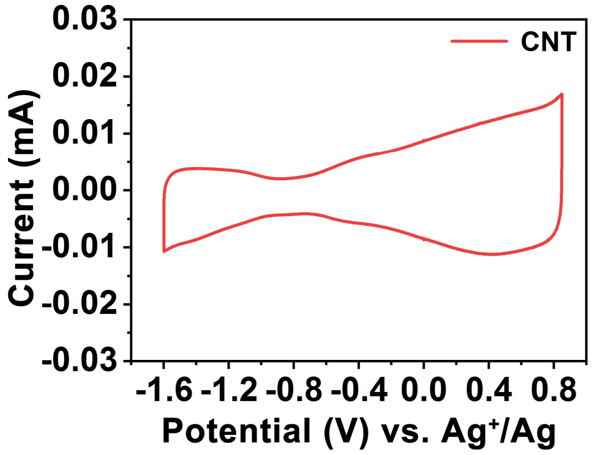

Figure S4. CV curve of CNT at a scan rate of 0.5 mV s^{-1}.

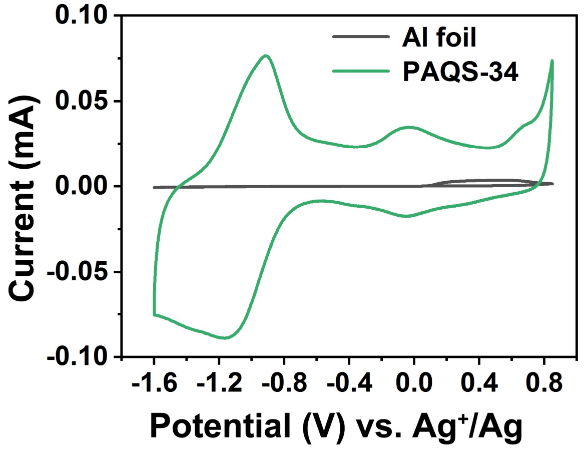

Figure S5. CV curves of Al foil and PAQS-34 at 0.5 mV s^{-1}.

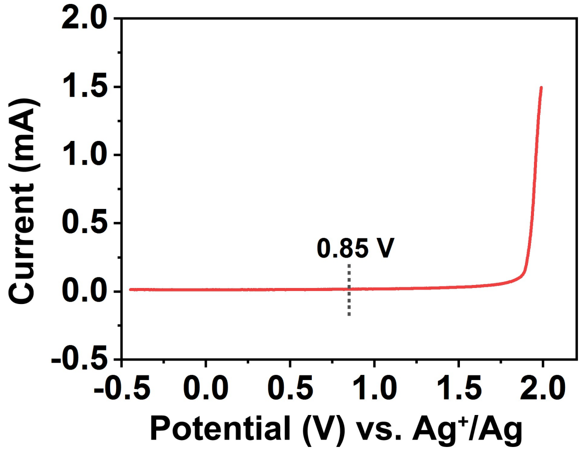

Figure S6. LSV of $Ca(TFSI)_2$ electrolyte at a scan rate of 10 mV s^{-1}.

LSV test conditions are as follows. Al foil as working electrode, active carbon as counter electrode and Ag^+/Ag as reference electrode. The scan rate is 10 mV s^{-1}.

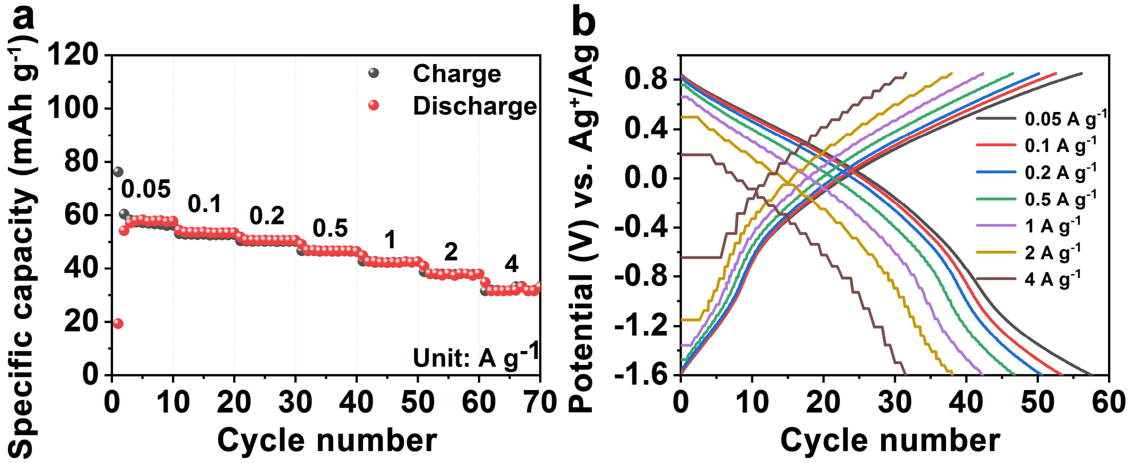

Figure S7. (a) Rate performance of CNT at different current densities. (b) GCD curves of CNT at different current densities.

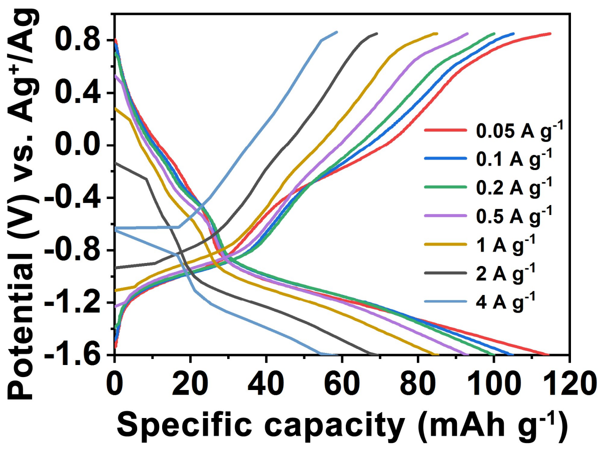

Figure S8. GCD curves of PAQS-34 at different current densities.

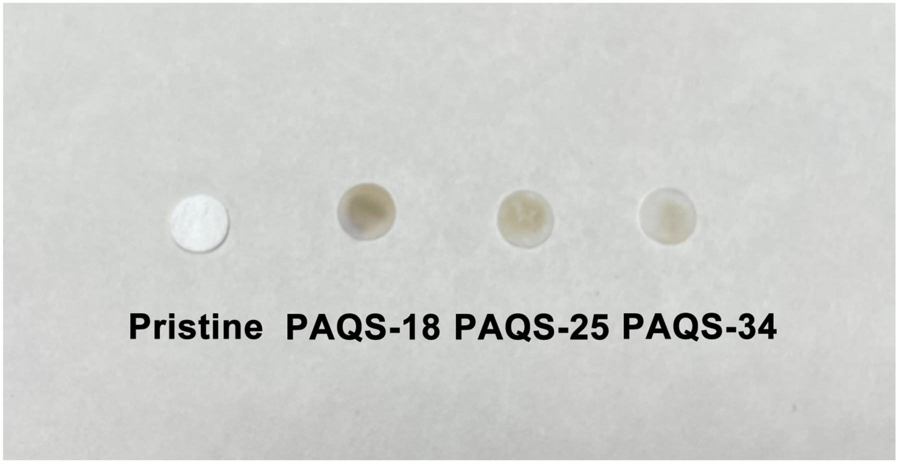

Figure S9. Membrane of pristine, PAQS-18, PAQS-25 and PAQS-34 after 500 cycles.

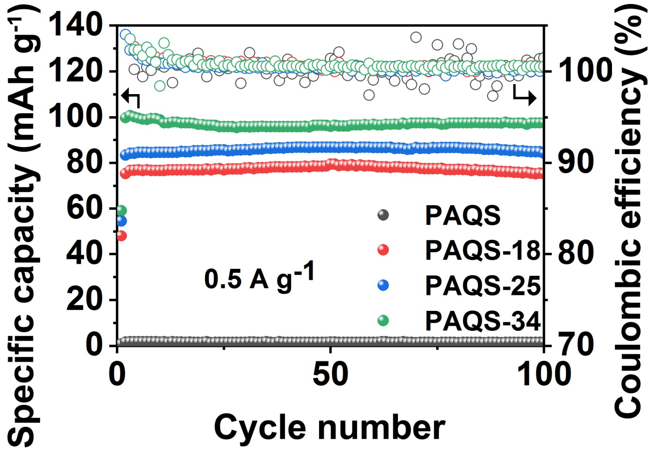

Figure S10. Long-term cycle performance of PAQS, PAQS-18, PAQS-25 and PAQS-34 at a current density of 0.5 A g^{-1} for 100 cycles.

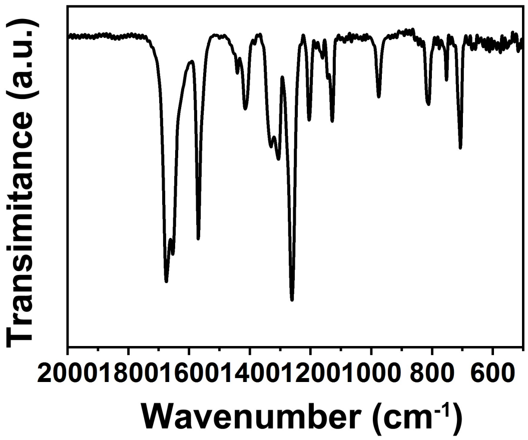

Figure S11. FTIR spectra of PAQS-41.

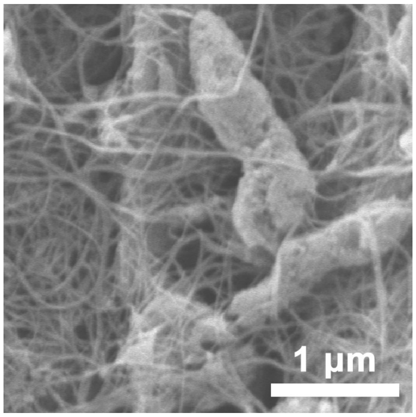

Figure S12. SEM image of PAQS-41.

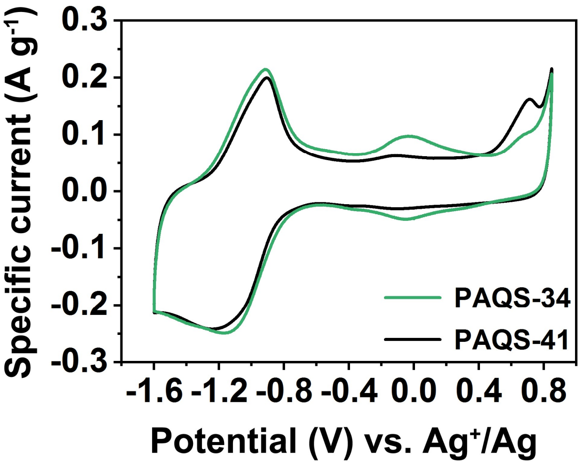

Figure S13. CV curves of PAQS-34 and PAQS-41 at 0.5 mV s^{-1}.

In Figure S19, higher CNT content shows lower charge-transfer resistance, which accelerates electron transport in the PAQS. In addition, more uniform dispersion in PAQS-34 leads to stronger interaction between the PAQS and CNT (Figure 2d). More complete conductive network enhances the electrochemical reaction kinetics. Because PAQS-34 shows the lowest charge-transfer resistance in all samples, the intensity of peak around 0.75 V is the lowest. However, excess CNT will be hard to disperse leading to agglomeration in the PAQS-41 (Figure S12), which results in the charge-transfer resistance of PAQS-41 larger than PAQS-34. So the peak around 0.75 V grows again for PAQS-41.

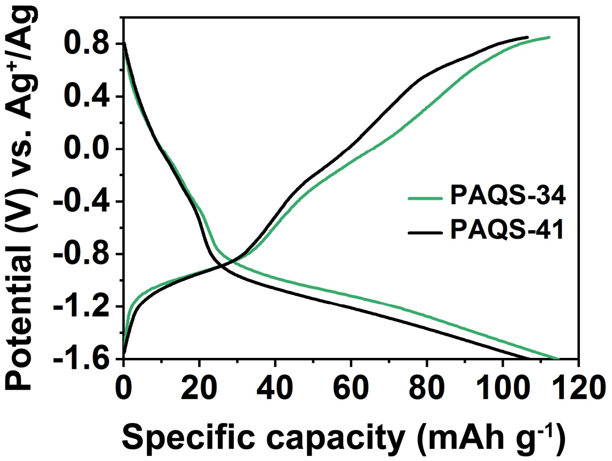

Figure S14. GCD curves of PAQS-34 and PAQS-41 at a current density of 0.1 A g^{-1} in the voltage range of -1.6-0.85 V vs. Ag^{+}/Ag.

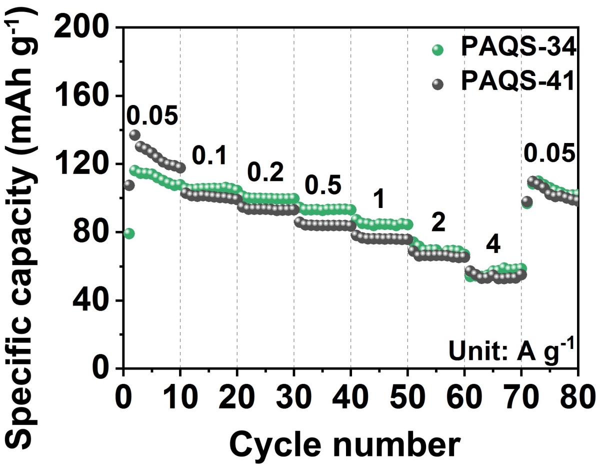

Figure S15. Rate performance of PAQS-34 and PAQS-41 at different current densities.

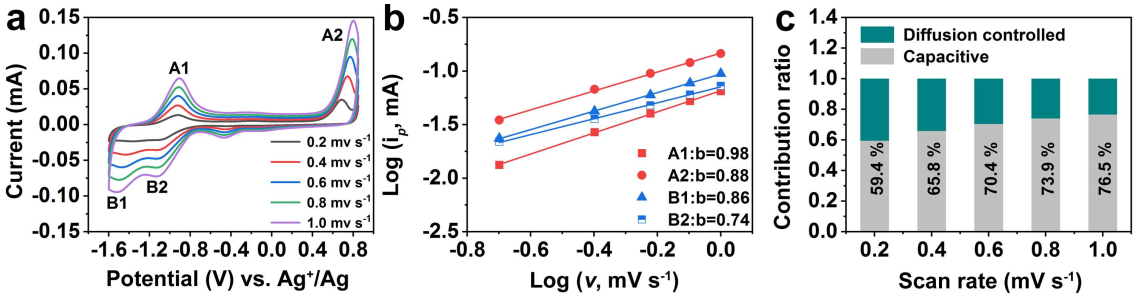

Figure S16. (a) CV curves of PAQS-18 at scanning rates from 0.2 to 1.0 mV s^{-1}. (b) Log (*v*) vs. Log (*i*) plots of PAQS-18. (c) Contribution ratio of the capacitive- and diffusion-controlled process of PAQS-18 at various scan rates.

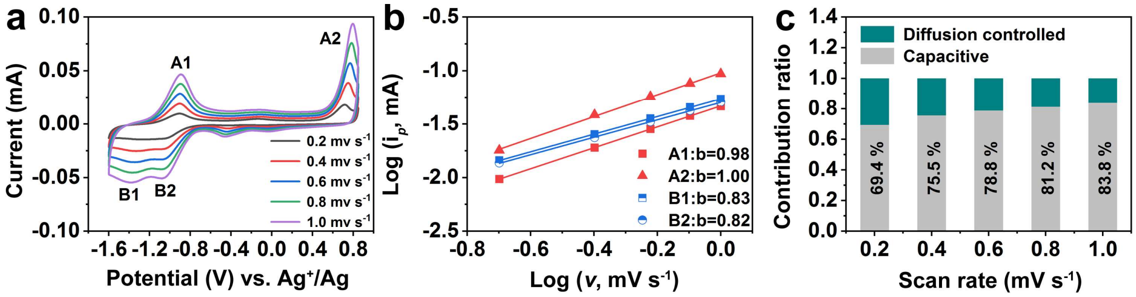

Figure S17. (a) CV curves of PAQS-25 at scanning rates from 0.2 to 1.0 mV s^{-1}. (b) Log (*v*) vs. Log (*i*) plots of PAQS-25. (c) Contribution ratio of the capacitive- and diffusion-controlled process of PAQS-25 at various scan rates.

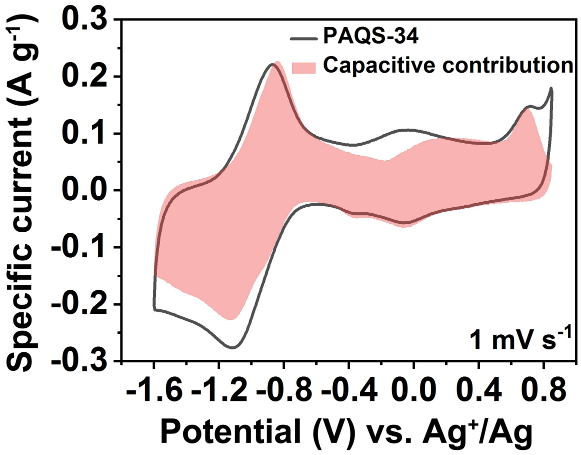

Figure S18. The capacitive contribution to the total current of PAQS-34 at 1 mV s^{-1}.

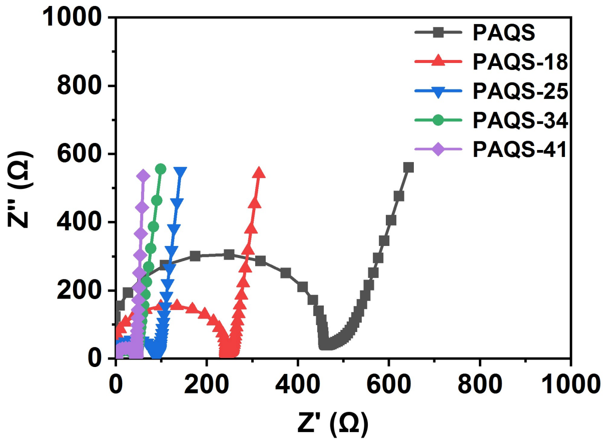

Figure S19. EIS curves of PAQS, PAQS-18, PAQS-25, PAQS-34 and PAQS-41.

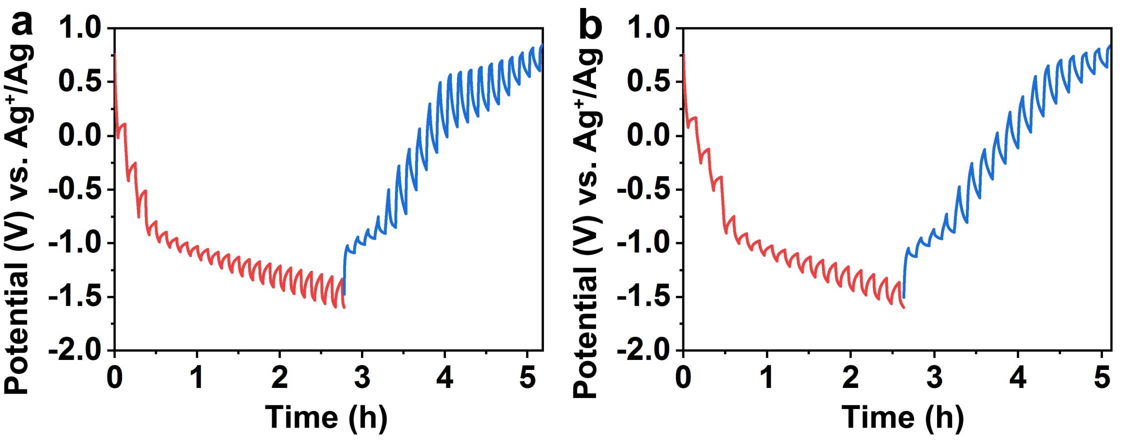

Figure S20. GITT curves of PAQS-18 (a) and PAQS-25 (b) during discharge and charge at a current density of 0.1 A g^{-1}.

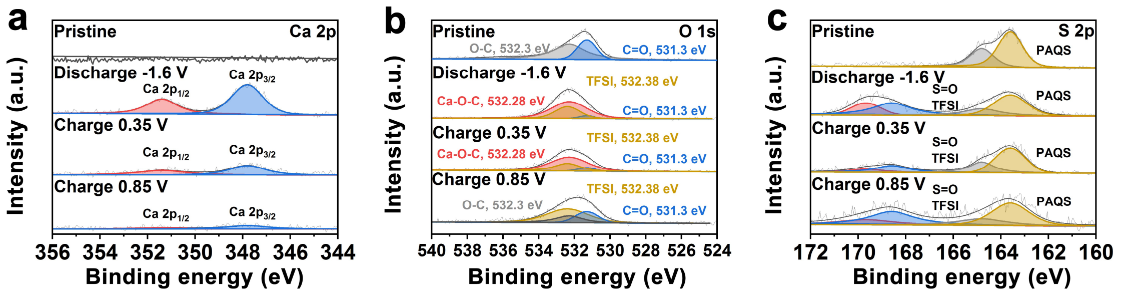

Figure S21. Ex situ XPS spectrum of PAQS-18 (a) Ca 2p. (b) O 1s. (c) S 2p.

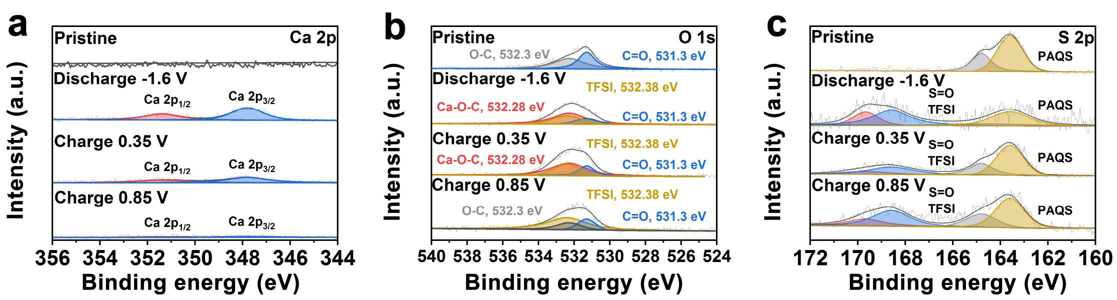

Figure S22. Ex situ XPS spectrum of PAQS-25 (a) Ca 2p. (b) O 1s. (c) S 2p.

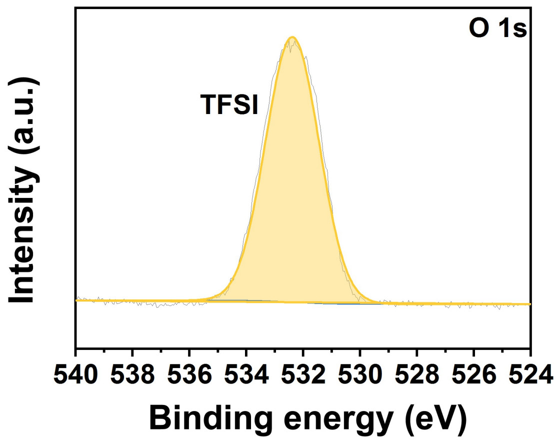

Figure S23. XPS O 1s spectrum of $Ca(TFSI)_2$ powder.

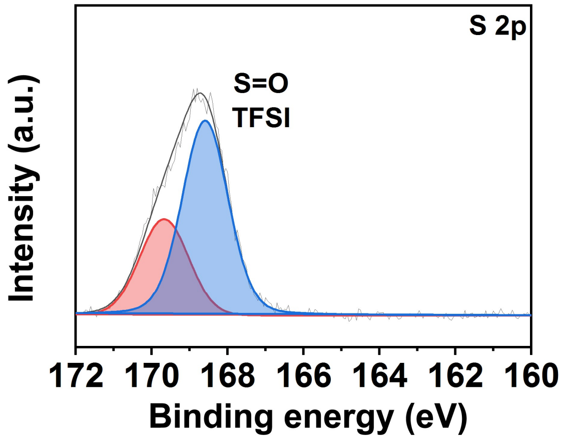

Figure S24. XPS S 2p spectrum of $Ca(TFSI)_2$ powder.

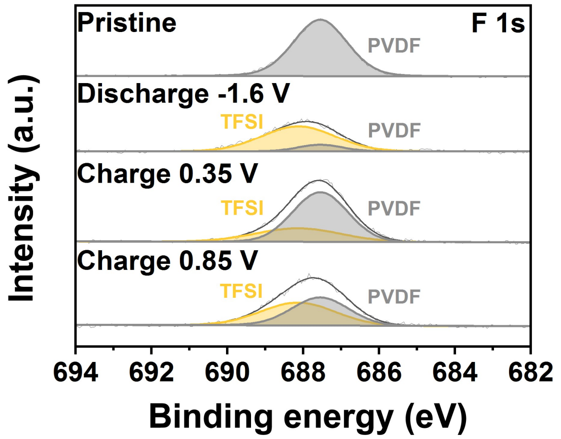

Figure S25. Ex situ XPS F 1s spectrum of PAQS-34.

The pristine state of PAQS-34 shows F 1s at 687.5 eV from PVDF. However, the new peak of F 1s from $TFSI^-$ anion appears at 688.1 eV at the discharged state, which also suggests the co-insertion of $Ca(TFSI)^+$ in the discharging process. The XPS F 1s spectra of $Ca(TFSI)_2$ powder are shown in Figure S26, which proves that the new peak is F 1s from $TFSI^-$ anion. After charged to 0.35 V vs. Ag^+/Ag, the peak intensity of $TFSI^-$ was decreased because $Ca(TFSI)^+$ was removed from PAQS-34 during the charging process. When charged to 0.85 V vs. Ag^+/Ag, the peak intensity of $TFSI^-$ was increased again, which is due to the intercalation of $TFSI^-$ into CNT during high potential.

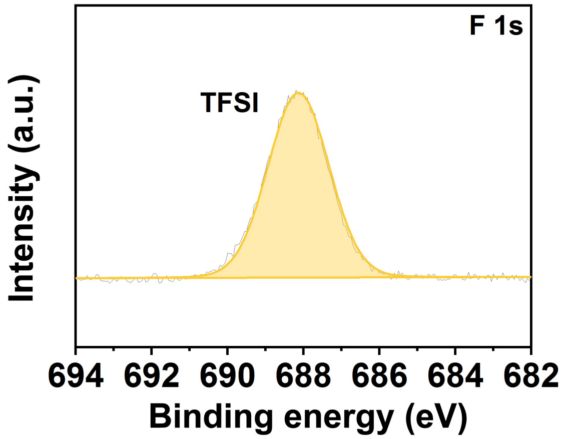

Figure S26. XPS F 1s spectrum of $Ca(TFSI)_2$ powder.

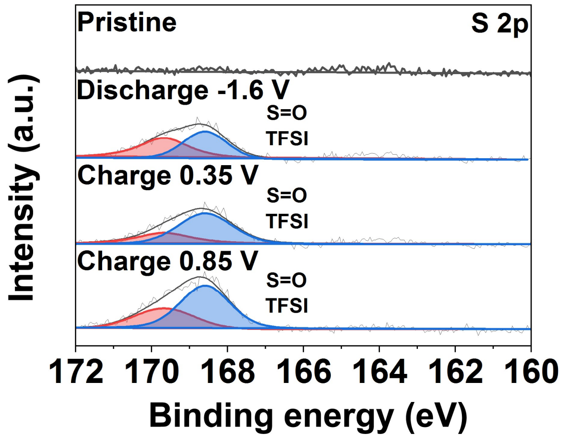

Figure S27. Ex situ XPS S 2p spectrum of CNT.

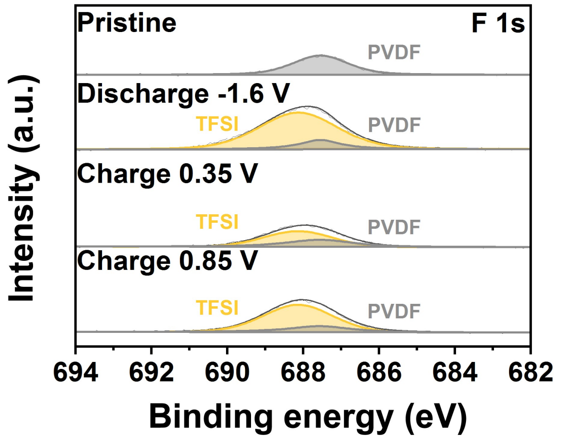

Figure S28. Ex situ XPS F 1s spectrum of CNT.

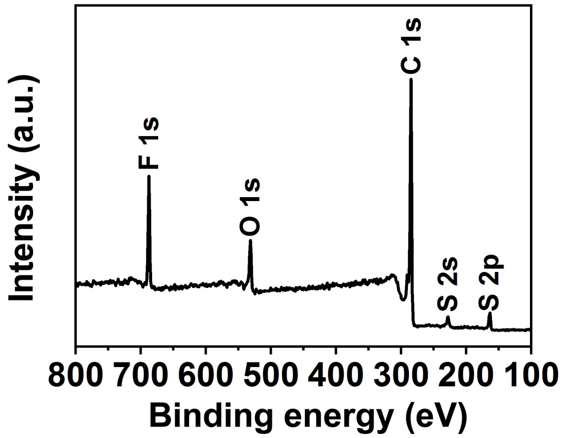

Figure S29. Full survey XPS spectrum of PAQS-34 electrode.

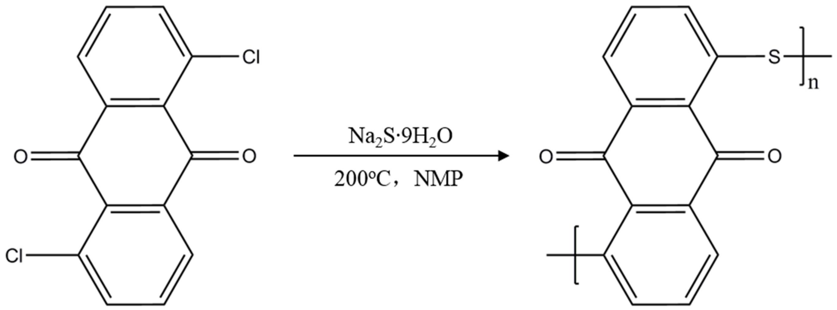

Figure S30. Synthetic structure diagram of PAQS.

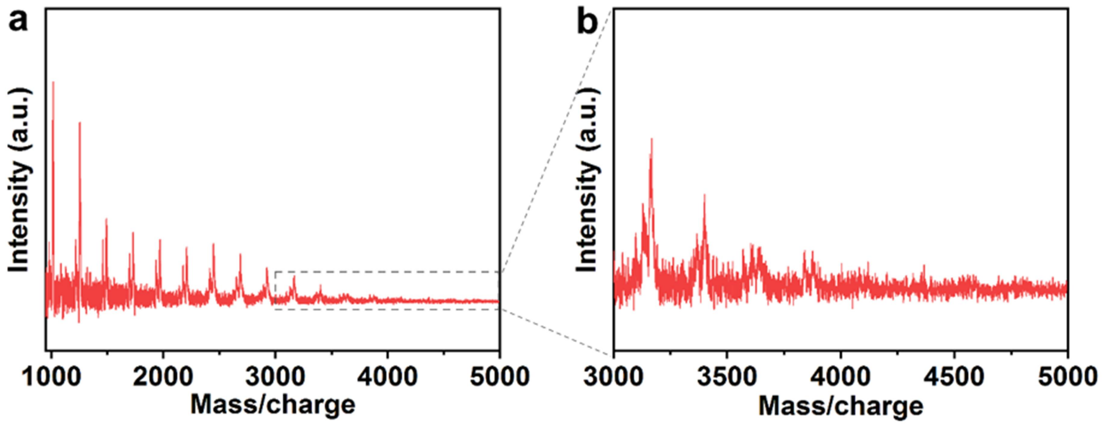

Figure S31. MALDI-TOF mass spectra for PAQS. (a) Full spectrum. (b) Zoomed-in spectrum for mass/charge = 3000-5000.

The MALDI-TOF mass spectra of PAQS show two adjacent peaks. The difference of Mass/charge between the two adjacent peaks is 35.45. This is because one of the chlorine atoms on the edge of the polymer is removed by Na_2S but it does not continue to connect more monomers.

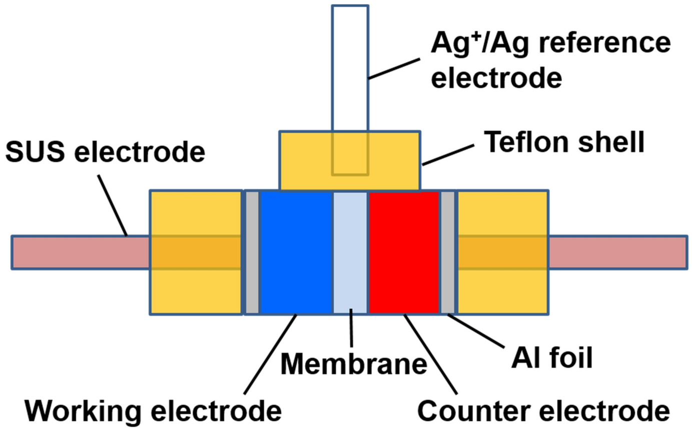

Figure S32. Structure diagram of three-electrode battery (Swagelok).

Table S1. Energy value of each component for step 1.

Specice	E (Hatree)
$Ca(TFSI)_2$	-4332.7432361
PAQS	-1126.3036597
AC	-615.7387323
$PAQS\text{-}Ca(TFSI)^+$	-3631.5453457
$AC\text{-}TFSI^-$	-2443.3594739

ΔE= -0.1191915 Hatree = -312.93728325 kJ/mol

1 Hatree = 2625.5 kJ/mol

Table S2. Energy value of each component for step 2.

Specice	E (Hatree)
PAQS-Ca(TFSI)$^{+}$	-3631.5453457
AC	-615.7387323
PAQS-Ca^{2+}	-1803.9354486
AC-TFSI^{-}	-2443.3594739

ΔE= -0.0108445 Hatree = -28.47223475 kJ/mol

1 Hatree = 2625.5 kJ/mol

Table S3. Atomic content of the detected elements obtained by XPS for PAQS-34.

PAQS-34	Ca	N	C	S	O	F	Active N	Ca/Active N
Pristine	0	1.76	77.79	3.06	7.62	9.78	0	-
Discharge -1.6 V	3.11	3.3	58.73	3.25	21.59	10.02	1.54	2.02
Charge 0.35 V	1.22	2.87	69.84	3.43	10.97	11.68	1.11	1.09
Charge 0.85 V	0.51	3.53	67.97	3.96	12.13	11.91	1.77	0.28

The N content of pristine PAQS-34 is derived from CNT including slight N atom.

Table S4. Performance comparison with reported cathode materials for non-aqueous CIBs.

Materials	Current Density (mA g^{-1})	Maximum discharge capacity (mAh g^{-1})	Cycle number, retained capacity	Reference
PAQS-34	50	116	-	This work
	100	106	-	
	200	101	-	
	500	95	100 cycles, 94 mAh g^{-1}	
	1000	87	500 cycles, 69 mAh g^{-1}	
	2000	74	-	
	4000	59	-	
$Mg_{0.25}V_2O_5{\cdot}H_2O$	20	122	20 cycles, 110 mAh g^{-1}	[4]
	50	91	100 cycles, 97 mAh g^{-1}	
	100	70	500 cycles, 61 mAh g^{-1}	
$Na_xMnFe(CN)_6$	10	100	35 cycles, ~50 mAh g^{-1}	[5]

$K_2BaFe(CN)_6$	12.5	60	30 cycles, 55.8 mAh g^{-1}	[6]
$CaCo_2O_4$	40	93	30 cycles, ~80 mAh g^{-1}	[7]
Na_2FePO_4F	10	60	12 cycles, ~42 mAh g^{-1}	[8]
$Fe_4[Fe(CN)_6]_3$	23	150	-	[9]
	125	120	80 cycles, ~103 mAh g^{-1}	
Na-doped $NH_4V_4O_{10}$	100	153	100 cycles, ~150 mAh g^{-1}	[10]
	300	92	-	
	500	72	-	
	1000	61	-	
$VOPO_4·2H_2O$	10	118	-	[11]
	20	100	35 cycles, ~86 mAh g^{-1}	
	50	73	-	

	100	57	200 cycles, ~50 mAh g^{-1}	
	200	42.7	-	
$Ag_{0.33}V_2O_5$	12.3	179	50 cycles, 85 mAh g^{-1}	[12]
	24.6	120	-	
	61.5	90	-	
	123	30	-	
$NaV_2(PO_4)_3$	3.5	81	40 cycles, 83 mAh g^{-1}	[13]
$FePO_4$	7.5	72	25 cycles, ~30 mAh g^{-1}	
$K_{0.5}V_2O_5$	13.3	100	50 cycles, ~55 mAh g^{-1}	[14]
	40	87	-	
	66.5	70	100 cycles, 60 mAh g^{-1}	
	133.1	50	-	

	20	303	-	
	50	200	-	
$FeV_3O_9{\cdot}1.2H_2O$	100	140	-	[15]
	200	96	400 cycles, 83 mAh g^{-1}	

References

[1] a) B. Delley, J. Chem. Phys. **1990**, 92, 508; b) B. Delley, J. Chem. Phys. **2000**, 113, 7756.

[2] J. P. Perdew, Y. Wang, Phys. Rev. B **1992**, 45, 13244.

[3] Z.-L. Xu, J. Park, J. Wang, H. Moon, G. Yoon, J. Lim, Y.-J. Ko, S.-P. Cho, S.-Y. Lee, K. Kang, Nat. Commun. **2021**, 12.

[4] X. Xu, M. Duan, Y. Yue, Q. Li, X. Zhang, L. Wu, P. Wu, B. Song, L. Mai, ACS Energy Lett. **2019**, 4, 1328.

[5] A. L. Lipson, B. Pan, S. H. Lapidus, C. Liao, J. T. Vaughey, B. J. Ingram, Chem. Mater. **2015**, 27, 8442.

[6] P. Padigi, G. Goncher, D. Evans, R. Solanki, J. Power Sources **2015**, 273, 460.

[7] M. Cabello, F. Nacimiento, J. R. González, G. Ortiz, R. Alcántara, P. Lavela, C. Pérez-Vicente, J. L. Tirado, Electrochem. Commun. **2016**, 67, 59.

[8] A. L. Lipson, S. Kim, B. Pan, C. Liao, T. T. Fister, B. J. Ingram, J. Power Sources **2017**, 369, 133.

[9] N. Kuperman, P. Padigi, G. Goncher, D. Evans, J. Thiebes, R. Solanki, J. Power Sources **2017**, 342, 414.

[10] T. N. Vo, H. Kim, J. Hur, W. Choi, I. T. Kim, J. Mater. Chem. A **2018**, 6, 22645.

[11] J. Wang, S. Tan, F. Xiong, R. Yu, P. Wu, L. Cui, Q. An, Chem. Commun. **2020**, 56, 3805.

[12] J. Hyoung, J. W. Heo, B. Jeon, S.-T. Hong, J. Mater. Chem. A **2021**, 9, 20776.

[13] S. Kim, L. Yin, M. H. Lee, P. Parajuli, L. Blanc, T. T. Fister, H. Park, B. J. Kwon, B. J. Ingram, P. Zapol, R. F. Klie, K. Kang, L. F. Nazar, S. H. Lapidus, J. T. Vaughey, ACS Energy Lett. **2020**, 5, 3203.

[14] M. E. Purbarani, J. Hyoung, S.-T. Hong, ACS Appl. Energy Mater. **2021**, 4, 7487.

[15] M. S. Chae, D. Setiawan, H. J. Kim, S.-T. Hong, Batteries **2021**, 7, 54.
